# JNK activation is essential for activation of MEK/ERK signaling in IL-1β-induced COX-2 expression in synovial fibroblasts

**DOI:** 10.1038/srep39914

**Published:** 2017-01-05

**Authors:** Taku Kitanaka, Rei Nakano, Nanako Kitanaka, Taro Kimura, Ken Okabayashi, Takanori Narita, Hiroshi Sugiya

**Affiliations:** 1 Laboratory of Veterinary Biochemistry, Department of Veterinary Medicine, College of Bioresource Sciences, Nihon University, 1866 Kameino, Fujisawa, Kanagawa, Japan; 2 Kimura Animal Hospital, 50 Babashitacho, Shinjuku, Tokyo, Japan

## Abstract

The proinflammatory cytokine interleukin 1β (IL-1β) induces prostaglandin E_2_ (PGE_2_) production via upregulation of cyclooxygenase-2 (COX-2) expression in synovial fibroblasts. This effect of IL-1β is involved in osteoarthritis. We investigated MAPK signaling pathways in IL-1β-induced COX-2 expression in feline synovial fibroblasts. In the presence of MAPK inhibitors, IL-1β-induced COX-2 expression and PGE_2_ release were both attenuated. IL-1β induced the phosphorylation of p38, JNK, MEK, and ERK1/2. A JNK inhibitor prevented not only JNK phosphorylation but also MEK and ERK1/2 phosphorylation in IL-1β-stimulated cells, but MEK and ERK1/2 inhibitors had no effect on JNK phosphorylation. A p38 inhibitor prevented p38 phosphorylation, but had no effect on MEK, ERK1/2, and JNK phosphorylation. MEK, ERK1/2, and JNK inhibitors had no effect on p38 phosphorylation. We also observed that in IL-1β-treated cells, phosphorylated MEK, ERK1/2, and JNK were co-precipitated with anti-phospho-MEK, ERK1/2, and JNK antibodies. The silencing of JNK1 in siRNA-transfected fibroblasts prevented IL-1β to induce phosphorylation of MEK and ERK1/2 and COX-2 mRNA expression. These observations suggest that JNK1 phosphorylation is necessary for the activation of the MEK/ERK1/2 pathway and the subsequent COX-2 expression for PGE_2_ release, and p38 independently contributes to the IL-1β effect in synovial fibroblasts.

Osteoarthritis (OA) is characterized by pain, swelling, and stiffness of articulations due to an alteration and loss of articular cartilage. This process is the result of pathologic cellular changes in bone, cartilage, ligaments, and synovium. Cartilage degeneration has been considered as a major sign of OA, but it is now recognized that synovitis, inflammation of synovial membrane, plays a crucial role in early and late stage of OA^1^,^2^. Inflammatory mediators involved in synovitis attract leukocytes into the joint and degrade the extracellular matrix^3^,^4^. Synovial fibroblasts have the potential to synthesize and release inflammatory mediators such as interleukin-1β (IL-1β), IL-6, IL-8, and prostaglandins including prostaglandin E_2_^3^,^5^. Prostaglandin E_2_ is considered the major contributor to inflammatory pain in arthritic conditions^6^ as the increase in prostaglandin E_2_ level was observed in synovial fluid of human with osteoarthritis and the canine osteoarthritis model^7^,^8^,^9^. Furthermore, the suppression of prostaglandin E_2_ production by non-steroidal anti-inflammatory drugs, such as meloxicam, is provided to relief the chronic pain in animals with osteoarthritis^10^,^11^.

IL-1β, a cytokine involved in the inflammatory response, induces prostaglandin E_2_ synthesis via cyclooxygenase-2 (COX-2) expression in proinflammatory states^6^,^10^,^11^,^12^. It has been reported that IL-1β activates several cellular signaling pathways including Mitogen-activated Protein Kinase (MAPK) signaling.

MAPK signaling pathways are involved in the regulation of various cellular functions including inflammation^13^,^14^. MAPKs are serine-threonine kinases and include c-Jun NH_2_-terminal kinase (JNK), p38 MAPK, and extracellular signal-regulated kinase (ERK); all of them exist in several isoforms, in mammals. The activation of these MAPKs is induced through different pathways, depending on the stimulus and the cell type, resulting in specific cellular responses through the phosphorylation of a wide range of substrates such as transcription factors and cytoskeletal proteins^13^,^14^,^15^.

It is assessed that MAPK signaling cascades consist of at least three hierarchically sequential kinase components: a MAPK kinase kinase (MAPKKK), a MAPK kinase (MAPKK), and a MAPK. MAPKKKs activate MAPKKs through phosphorylation on serine or threonine residues, which in turn activate MAPKs through phosphorylation of both threonine and tyrosine residues in its activation loop^16^,^17^.

We investigated IL-1β-induced COX-2 expression and its role in the synthesis of prostaglandin E_2_ in feline synovial fibroblasts. Our study found a cross-talk regulation between different MAPK signaling pathways. Moreover, we demonstrate that JNK regulates MEK/ERK signaling in IL-1β-induced synovial fibroblasts.

## Results

### Characterization of IL-1β-induced prostaglandin E_2_ release via COX-2 expression in feline synovial fibroblasts

**Figure 1:**
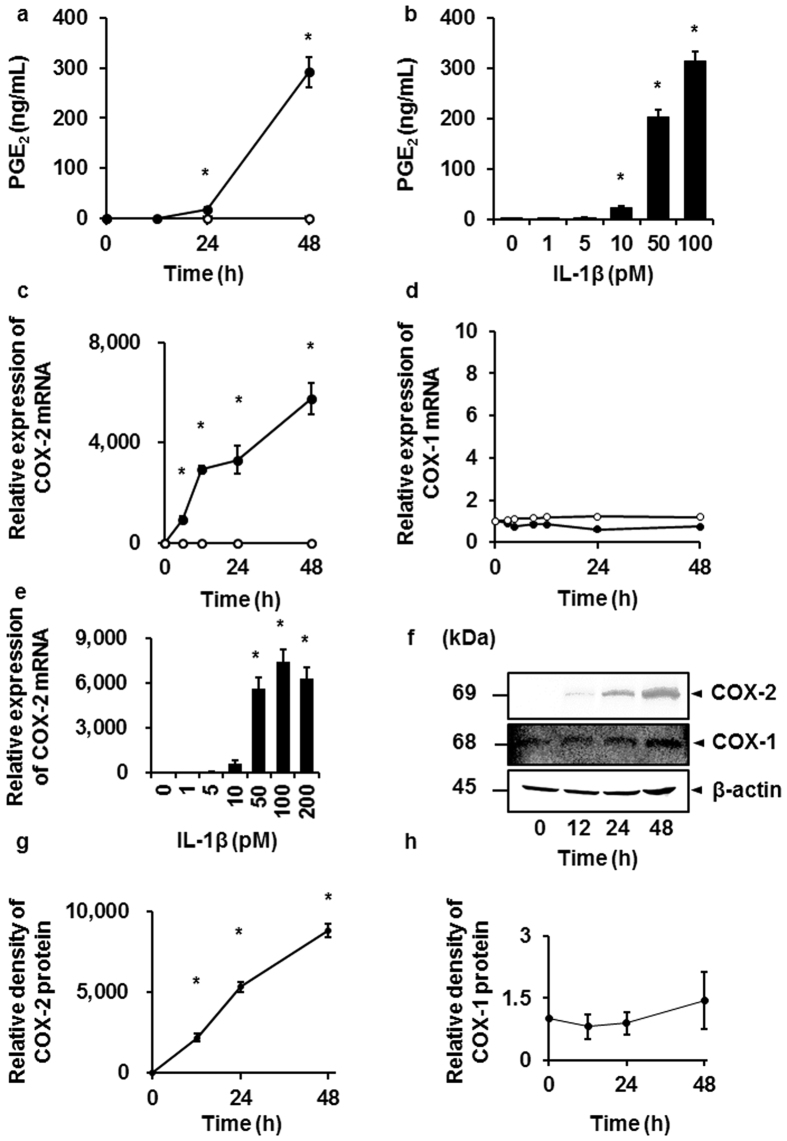
IL-1β-induced prostaglandin E_2_ release and COX-2 mRNA and protein expression in feline synovial fibroblasts. When cells were treated with (closed circle) or without (open circle) feline recombinant IL-1β (50 pM), prostaglandin E_2_ (PGE_2_) release (**a**) and COX-2 mRNA expression (**c**) were increased in a time-dependent manner. When cells were treated with the indicated concentrations of IL-1β for 48 h, PGE_2_ release (**b**) and COX-2 mRNA expression (**d**) were stimulated in a dose-dependent manner. IL-1β had no effect on COX-1 mRNA expression (**e**). In cells treated with IL-1β (50 pM) for 0–48 h, COX-2 (**f**; first row), COX-1 (**f**; second row) and β-actin (**f**; third row) protein expression was examined. Relative density of COX-2 (**g**) compared with that time 0 was provoked in a time-dependent manner, whereas IL-1β had no effect on COX-1 protein expression (**h**). Results are presented as mean ± SE from 3 independent experiments. The F values were 99.12 (**a**), 197.50 (**b**), 33.69 (**c**), 69.95 (**e**) and 440.67 (**g**). The degrees of freedom were 3 (**a**), 5 (**b**), 8 (**c**), 6 (**e**) and 3 (**g**). *P < 0.05, compared with 0 h (**a,c,g**) or 0 pM (**b,e**).

In various kinds of cells such as dermal fibroblasts, IL-1β induces prostaglandin E_2_ release via COX-2 expression^18^,^19^,^20^,^21^,^22^,^23^. Therefore, the first step in our work was the characterization of IL-1β-induced prostaglandin E_2_ release and COX expression in feline synovial fibroblasts. The treatment of synovial fibroblasts with IL-1β (50 pM) induced prostaglandin E_2_ release in a time-dependent manner (Fig. 1a). The incubation of cells with IL-1β for 48 h stimulated prostaglandin E_2_ release in a dose-dependent manner (Fig. 1b). The conversion of arachidonic acid into prostaglandin E_2_ is mediated by two isoforms of COX, COX-1, and COX-2, which are constitutive and inducible forms, respectively^18^,^20^. Subsequently, we examined the effect of IL-1β on COX mRNA expression. As Fig. 1c and e summarize, IL-1β increased COX-2 mRNA expression in a time- and dose-dependent manner, respectively, but had no effect on COX-1 mRNA expression (Fig. 1d). Moreover, in the cells treated with IL-1β, COX-2 protein expression increased time-dependently (Fig. 1f,g). However, there is no significant difference in COX-1 protein expression in IL-1β-treated feline synovial fibroblasts (Fig. 1f,h). Taken together, it is most likely that IL-1β stimulates prostaglandin E_2_ release via COX-2 expression in feline synovial fibroblasts.

### Involvement of MEK, ERK1/2, JNK and p38 in IL-1β-induced COX-2 mRNA expression

**Figure 2:**
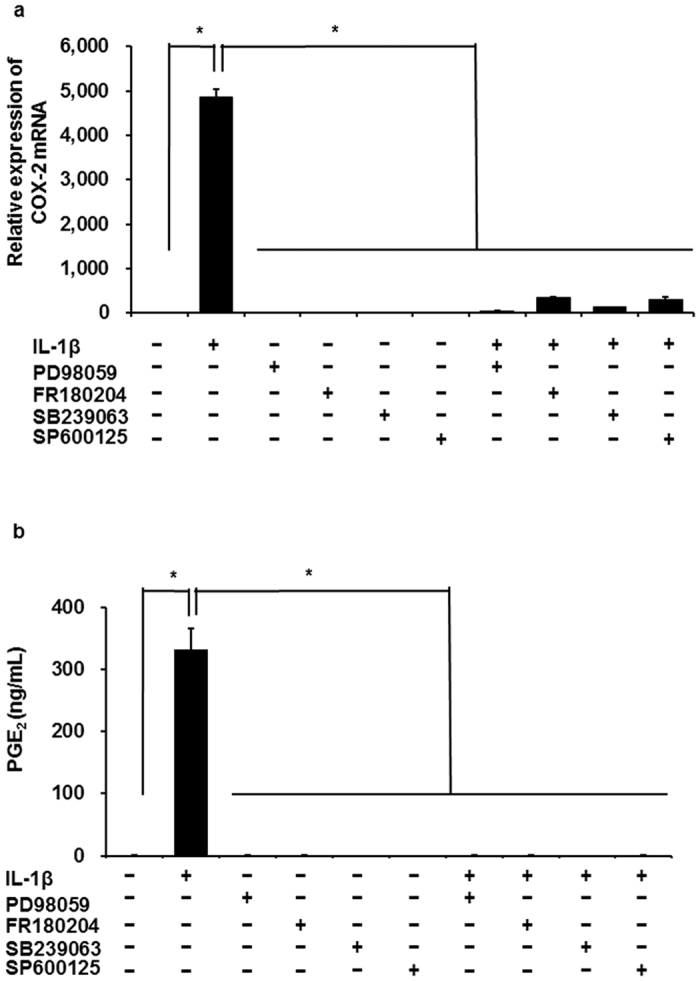
Effect of MEK, ERK1/2, JNK, and p38 inhibitors on IL-1β-induced COX-2 mRNA expression. When synovial fibroblasts were pretreated with the MEK inhibitor PD98059 (50 μM), the ERK1/2 inhibitor FR180204 (50 μM), the JNK inhibitor SP600125 (10 μM) and the p38 inhibitor (20 μM) for 1 h, IL-1β-induced COX-2 mRNA expression (**a**) and prostaglandin E_2_ release (**b**) were significantly attenuated. Results are presented as mean ± SE from 3 independent experiments. The F values were 584.72 (**a**) and 92.64 (**b**). The degree of freedom was 9 (**a**,**b**). *P < 0.05.

In mammalian cells, MAPK signaling plays an important role in inflammation responses. Three MAPK signaling pathways have been clearly characterized: MEK/ERK1/2, JNK and p38 MAPK signaling pathways^13^,^14^. We examined the contribution of MAPK signaling Pathways to IL-1β-induced COX-2 expression in feline synovial fibroblasts by MAPK inhibitors. The IL-1β-induced COX-2 mRNA expression was clearly inhibited in the presence of the MEK inhibitor PD98059, the ERK1/2 inhibitor FR180204, the JNK inhibitor SP600125, or the p38 inhibitor SB239063 (Fig. 2a). These treatments also lead to a significant attenuation of IL-1β-induced prostaglandin E_2_ release (Fig. 2b).

**Figure 3:**
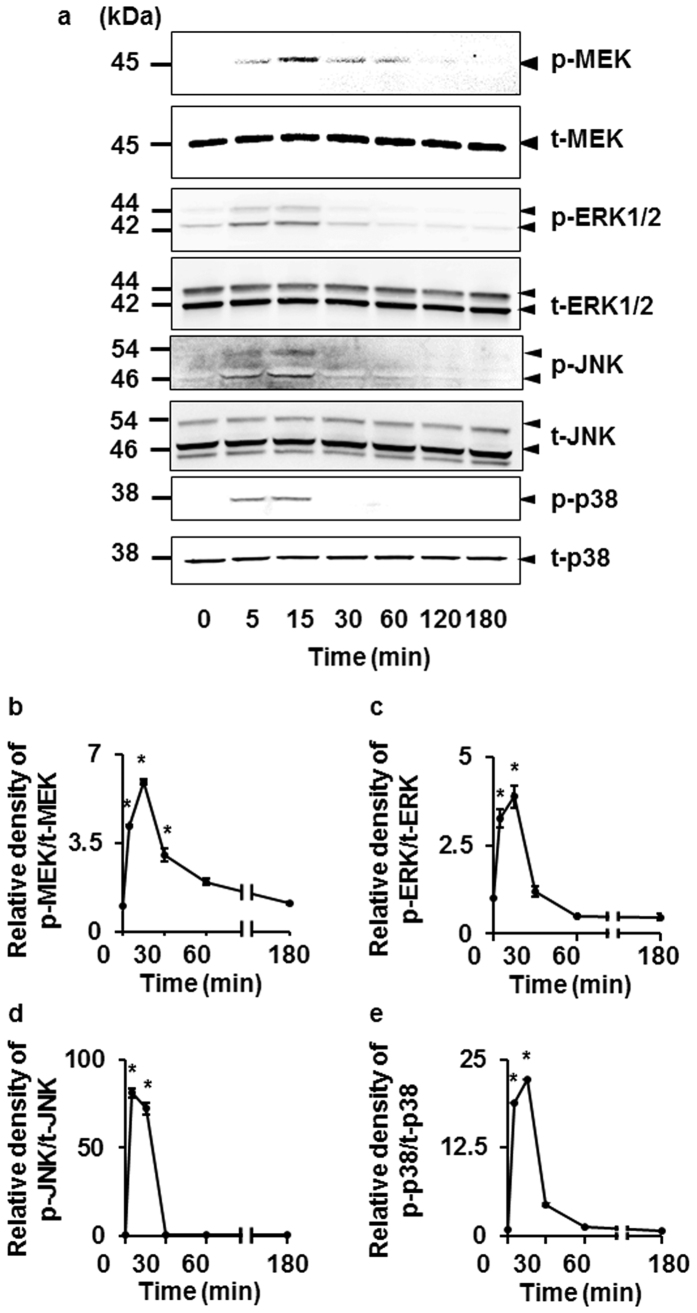
IL-1β-induced phosphorylation of MEK, ERK1/2, JNK, and p38. (**a**) In cells treated with IL-1β (50 pM), the levels of phosphorylated MEK (p-MEK), total MEK (t-MEK), phosphorylated ERK1/2 (p-ERK1/2), total ERK1/2 (t-ERK1/2), phosphorylated JNK (p-JNK), total JNK (t-JNK), phosphorylated p38 (p-p38) and total p38 (t-p38) were detected by Western blotting. Relative density of p-MEK (**b**), p-ERK1/2 (**c**), p-JNK (**d**) and p-p38 (**e**) compared with that at time 0 is described. Results are presented as mean ± SE from 3 independent experiments. The F values were 136.25 (**b**), 487.47 (**c**), 502.91 (**d**) and 97.77 (**e**). The degrees of freedom was 6 (**b–e**). *P < 0.05.

We next examined whether IL-1β induced the phosphorylation of MEK, ERK1/2, JNK, and p38. In cells treated with IL-1β, the phosphorylation of MEK, ERK1/2, JNK, and p38 occurred in a time-dependent manner. The peak of phosphorylation of each protein was observed at 15 min after IL-1β stimulation (Fig. 3). These results strongly suggest that the MEK/ERK1/2, JNK, and p38 signaling pathways are involved in IL-1β-induced COX-2 expression and prostaglandin E_2_ release in feline synovial fibroblasts.

### Attenuation of IL-1β-induced MEK and ERK1/2 phosphorylation by the JNK inhibitor

**Figure 4:**
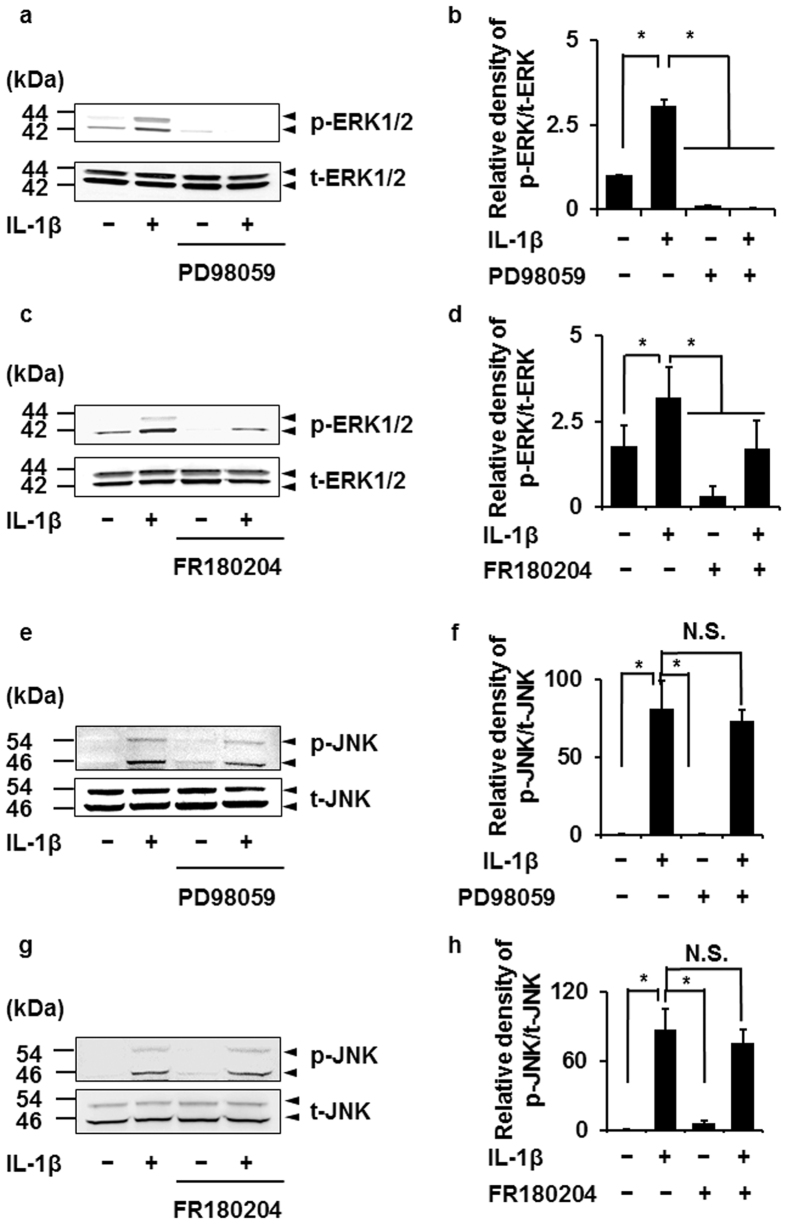
Effect of MEK and ERK1/2 inhibitors on IL-1β-induced phosphorylation of ERK1/2 and JNK. After pretreatment with the MEK inhibitor PD98059 (50 μM) and the ERK1/2 inhibitor FR180204 (50 μM) for 1 h, cells were stimulated with IL-1β for 15 min. MEK and ERK1/2 inhibitors attenuated IL-1β-induced phosphorylation of ERK1/2 (**a–d**) but not that of JNK (**e–h**). Relative density of p-ERK1/2 (**b,d**) and of p-JNK (**f,h**) compared with that of the absent of IL-1β is described. Results are presented as mean ± SE from 3 independent experiments. The F values were 278.37 (**b**), 17.93 (**d**), 18.26 (**f**) and 24.49 (**h**). The degrees of freedom was 3 (**b**,**d**,**f**,**h**). *P < 0.05.

**Figure 5:**
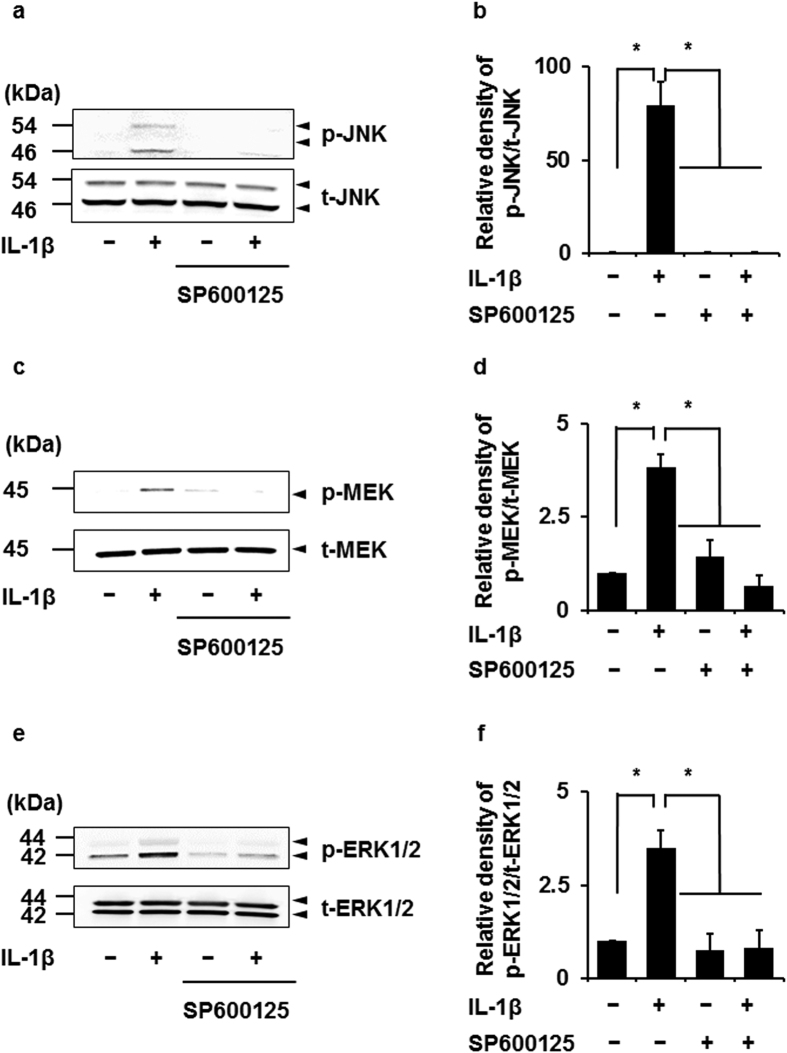
Effect of the JNK inhibitor on IL-1β-induced phosphorylation of JNK and ERK1/2. After pretreatment with the JNK inhibitor SP600125 (10 μM) for 1 h, cells were stimulated with IL-1β for 15 min. JNK inhibitor attenuated not only IL-1β-induced phosphorylation of JNK (**a,b**) but also that of MEK (**c,d**) and ERK1/2 (**e,f**). Relative density of p-JNK (**b**), p-MEK (**d**) and p-ERK1/2 (**f**) compared with that of the absent of IL-1β is described. Results are presented as mean ± SE from 3 independent experiments. The F values were 41.14 (**b**), 52.23 (**d**) and 11.80 (**f**). The degrees of freedom was 3 (**b**,**d**,**f**). *P < 0.05.

We examined the effect of MAPK inhibitors on p38, ERK1/2, and JNK phosphorylation induced by IL-1β. SB239063, PD98059, FR180204, and SP600125 were used as p38, MEK, ERK1/2, and JNK inhibitors, respectively. The p38 inhibitor clearly inhibited IL-1β-induced p38 phosphorylation, but had no effect on IL-1β-induced JNK and ERK1/2 phosphorylation (see Supplementary Fig. S1). The MEK and ERK1/2 inhibitors attenuated IL-1β-induced phosphorylation of ERK1/2 (Fig. 4a,b), but had no effect on that of p38 (see Supplementary Fig. S2) and JNK (Fig. 4e–h). The JNK inhibitor SP600125 clearly inhibited IL-1β-induced JNK phosphorylation (Fig. 5a,b), but had no effect on IL-1β-induced p38 phosphorylation (see Supplementary Fig. S2). The ERK inhibitor FR180204 attenuated ERK1/2 phosphorylation in human U138 glioblastoma cells and human A549 lung epithelial cells^24^,^25^. The JNK inhibitor SP600125 inhibited the phosphorylation of JNK but not ERK1/2 in rat alveolar epithelial cells and lung tissue^26^,^27^. These observations support that the treatment of ERK inhibitor or JNK inhibitor attenuates the IL-1β-induced phosphorylation of ERK1/2 and JNK in feline synovial fibroblasts. However, surprisingly, the JNK inhibitor significantly attenuated IL-1β-induced MEK (Fig. 5c,d) and ERK1/2 phosphorylation (Fig. 5e,f). These observations suggest that p38 signaling is independently activated, but JNK signaling interacts with MEK/ERK1/2 signaling in IL-1β-stimulated synovial fibroblasts.

### Interaction of JNK and MEK/ERK signaling in IL-1β-stimulated synovial fibroblasts

**Figure 6:**
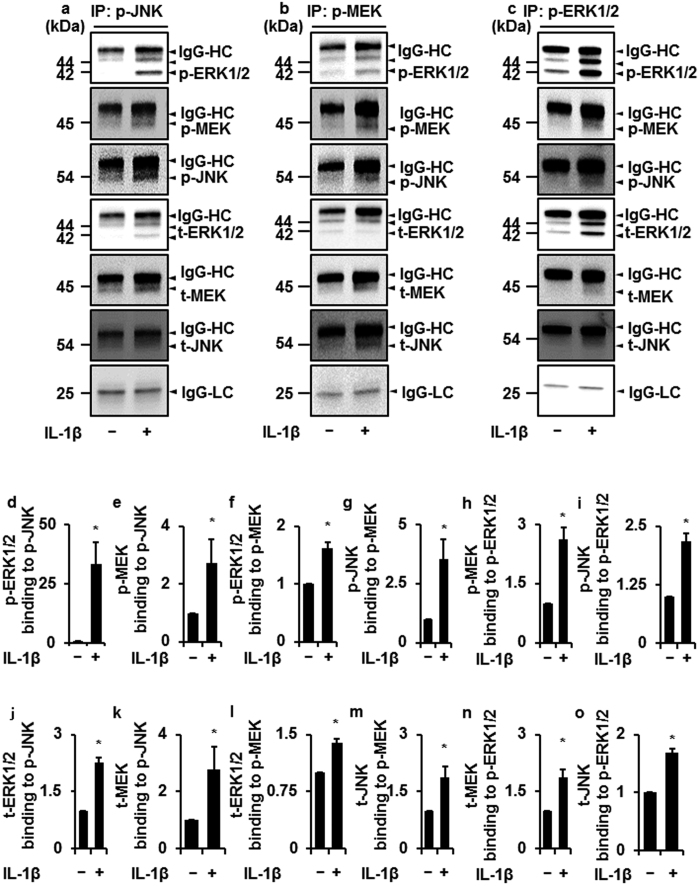
Interaction among JNK, MEK, and ERK1/2 in synovial fibroblasts treated with IL-1β. In fibroblasts treated with or without IL-1β, the fractions immunoprecipitated with anti-p-JNK (**a**,**d**,**e**,**j**,**k**), anti-p-MEK (**b**,**f**,**g**,**l**,**m**), and anti-p-ERK1/2 antibodies (**c**,**h**,**i**,**n**,**o**) were isolated; the levels of p-ERK1/2, p-MEK and p-JNK were detected by Western blotting. Relative density of p-ERK (**d**,**f**), p-MEK (**e**,**h**), p-JNK (**g**,**i**), t-ERK (**j**,**l**), t-MEK (**k**,**n**) and t-JNK (**m**,**o**) compared with that of the absent of IL-1β is described. For the immunoblotting, total cell lysate (100 μg protein) was used. IgG heavy chain (HC) and light chain (LC) were used as a loading control. There is no significant difference in IgG-HC and LC between control and IL-1β-treated cells. Results are presented as mean ± SE from 3 independent experiments. The T values were −3.64 (**d**), −2.96 (**e**), −5.69 (**f**), −3.05 (**g**), −5.11 (**h**), −4.28 (**i**), −10.23 (**j**), −2.23 (**k**), −7.24 (**l**), −3.07 (**m**), −6.94 (**n**) and −11.44 (**o**). The degree of freedom was 4 (**d–o**). *P < 0.05.

We examined the interaction between JNK and MEK/ERK1/2 by co-immunoprecipitation experiments. When feline synovial fibroblasts were stimulated with IL-1β, not only phosphorylated JNK but also phosphorylated MEK and ERK1/2 were detected in the fractions precipitated with anti-phospho-JNK antibody (Fig. 6a,d,e). Total MEK and ERK1/2 were also detected in the fractions precipitated with anti-phospho-JNK antibody (Fig. 6j,k). Similarly, total and phosphorylated MEK, ERK1/2 and JNK were detected in the fraction precipitated with anti-phospho-MEK and ERK1/2 antibodies in the cells stimulated with IL-1β (Fig. 6b,c,f–i,l–o). These observations suggest that the complex formation among JNK, MEK and ERK were induced following IL-1β treatment. Considering the outcomes of inhibitor experiments, it is likely that JNK activation evokes MEK/ERK1/2 activation and subsequently induces COX-2 expression in synovial fibroblasts stimulated with IL-1β.

**Figure 7:**
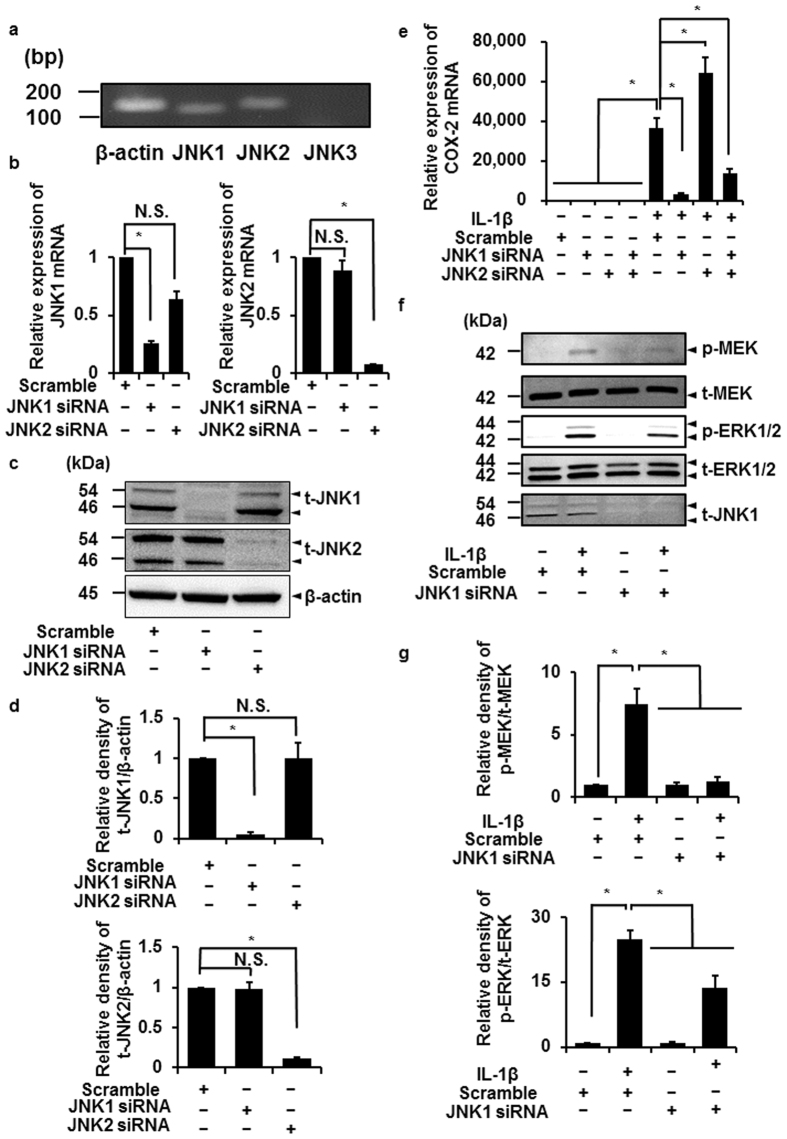
JNK-1 siRNA inhibits IL-1β-induced activation of MEK/ERK signaling. (**a**) JNK1 and JNK2 mRNA expression in feline synovial fibroblasts. Expression of three different subtypes of JNK was determined by RT-PCR using total RNA extracted from the fibroblasts. PCR products for JNK1 and JNK2 were detected to be 106 and 123 bp, respectively (**a**). mRNA and protein expression of JNK1 and JNK2 in JNK1 or JNK2 siRNA-transfected cells. JNK1 or JNK2 siRNA-transfection resulted in a significant decrease of expression of JNK-1 or JNK-2 mRNA (**b**) and protein (**c**), respectively, but not scramble siRNA transfection. Relative density of JNK-1 or JNK-2 protein expression in siRNA-transfected cells compared to that in scramble siRNA-transfected cells is illustrated (**d**). β-actin was used as an internal standard (**c**,**d**). Decrease in IL-1β-induced COX-2 mRNA expression in cells transfected with JNK1 siRNA but not in those transfected with JNK2 siRNA. JNK-1 siRNA transfection clearly inhibited the IL-1β-induced COX-2 mRNA expression compared with scramble siRNA transfection. The IL-1β-induced COX-2 mRNA expression was also attenuated in JNK1 and 2 double knockdown cells (**e**). Decrease in IL-1β-induced phosphorylation of MEK and ERK1/2 in JNK1 siRNA-transfected cells. In cells transfected JNK1 siRNA or scramble RNA, the levels of pMEK, t-MEK, p-ERK1/2, t-ERK1/2, and t-JNK1 were detected by Western blotting (**f**). Relative density of p-MEK/t-MEK and p-ERK/t-ERK compared to those with scramble siRNA transfection is illustrated (**g**). Results are presented as mean ± SE from 3 independent experiments. The F value were 82.76 (**b**; left panel), 128.85 (**b**; right panel), 21.87 (**d**; upper panel), 160.63 (**d**; lower panel), 51.31 (**e**), 21.34 (**g**; upper panel) and 58.45 (**g**; lower panel). The degrees of freedom were 2 (**b**,**d**), 7 (**e**) and 3 (**g**). *P < 0.05.

To confirm our hypothesis about the crucial role of JNK in the activation of the MEK/ERK1/2 pathway, we further performed JNK knockdown experiments using siRNA transfection. Currently three mammalian JNK genes are known to specify the JNK isoforms JNK1, JNK2, and JNK3^28^,^29^. Since mRNA expression of JNK1 and JNK2 isoforms was detected in feline synovial fibroblasts (Fig. 7a), we transfected fibroblasts with JNK1 or JNK2 siRNA. In these conditions, the expression of mRNA (Fig. 7b) and proteins (Fig. 7c,d) of JNK1 or JNK2 was significantly decreased, whereas transfection with a control scramble siRNA had no effect. In the JNK1-knockdown cells, IL-1β-induced COX-2 mRNA expression was significantly attenuated, whereas it was enhanced in the JNK2-knockdown cells (Fig. 7e). In the JNK1 and 2 double-knockdown cells, IL-1β-induced COX-2 mRNA expression was also inhibited (Fig. 7e). Then, we examined the effect of JNK1 knockdown on the IL-1β-induced phosphorylation of MEK and ERK1/2. In the fibroblasts transfected with JNK1 siRNA, IL-1β-induced phosphorylation of MEK and ERK1/2 was clearly inhibited, whereas it was observed in the fibroblasts transfected with scramble, as shown in Fig. 7f and g. These observations indicate that JNK signaling is upstream of MEK/ERK1/2 signaling and JNK1 activation needs IL-1β-induced MEK/ERK activation.

## Discussion

We demonstrated that the proinflammatory cytokine IL-1β induced COX-2 mRNA and protein expression along with prostaglandin E_2_ release in feline synovial fibroblasts. In the synovium, COX-2 expression was induced by stimuli such as IL-1β and TNF-α, which enhanced production of prostanoids including prostaglandin E_2_ that are involved in inflammatory responses^30^,^31^,^32^,^33^,^34^,^35^,^36^,^37^. Prostaglandin E_2_ produced by synovium has been suggested to lead to cartilage degradation, inhibition of matrix synthesis, and chondrocyte apoptosis^34^,^38^,^39^,^40^,^41^. Although COX-2 inhibitors are commonly prescribed to OA patients for pain relief and physical functioning, several studies have been reported that COX-2 selective inhibitors attenuated the development of OA^42^,^43^. In rat model, intra-articular injection of the selective COX-2 inhibitor meloxicam decreased the cartilage damage area and attenuated the nociceptive behaviors^43^. In human OA patients, buccal administration of the selective COX-2 inhibitor celecoxib decreased prostaglandin E_2_ release and resulted in the increase in proteoglycan content of cartilage^44^,^45^. These previous reports suggest that COX-2 expression in synovium plays a crucial role in OA pathogenesis via the production of prostaglandins. Therefore, it is possible that feline synovial fibroblasts stimulated by IL-1β are a model of synovitis in OA.

Multiple MAPK signaling pathways coordinate and integrate responses to diverse stimuli including cytokines such as IL-1β^13^,^14^. However, the response of MAPK signaling is highly dependent on the cellular context. In this study, we demonstrated that IL-1β induced the activation of p38, JNK and MEK/ERK1/2, and that the pharmacological inhibition of p38, JNK, MEK, and ERK1/2 completely attenuated IL-1β-induced COX-2 mRNA expression and prostaglandin E_2_ release. Together, these results suggest that the activation of p38, JNK, and MEK/ERK1/2 signaling plays a role in COX-2 expression and prostaglandin E_2_ release in synovial fibroblasts, which contributes to the pathogenesis of synovitis in OA.

We also demonstrated that the cross-talk between MAPK signaling in synovial fibroblasts stimulated with IL-1β. The most interesting finding of this study is that JNK inhibitor attenuated IL-1β-induced MEK and ERK1/2 phosphorylation, whereas MEK and ERK inhibitors had no effect on the JNK phosphorylation. It is generally accepted that the archetypal MAPK signaling pathway is composed of three hierarchically sequential kinase components^16^,^17^: a MAPKKK, a MAPKK, and a MAPK. MAPKKKs activate the downstream MAPKKs by phosphorylation, which in turn induce a phosphorylation-dependent increase in the activity of MAPKs. The MAPKs then elicit phosphorylation of various kinds of cytosolic or nuclear targets such as AP-1, which mediate the cellular responses to the original stimuli. ERK is well known to be activated by the MAPKK MEK. The phosphorylation and activation of MEK have been demonstrated to be elicited by MAPKKKs such as Raf^16^,^17^. However, our results suggest that JNK activation is necessary for the activation of the MEK/ERK1/2 signaling pathway in IL-1β-stimulated fibroblasts. In this study, SP600125 was used for preventing IL-1β-induced JNK activation. SP600125 was first reported as an ATP-competitive inhibitor for JNK, becasue this compound exhibited 10–100 fold selectivity for JNK over other protein kinases^46^. However, at a late time, many additional kinases (including MEK or other upstream kinases) have been reported to be targets of SP600125, because SP600125 bound and inhibited to a broad range of protein kinases with similar or greater potency than JNK^47^,^48^. On the other hand, antisense techniques or siRNA transfection has been introduced as one of the most specific way to inhibit JNK^49^. Therefore, we performed JNK-knockdown experiments by treatment with JNK subtype-specific siRNA. IL-1β-induced MEK/ERK1/2 phosphorylation and COX-2 mRNA expression were attenuated in the JNK1-knockdown cells. To confirm whether JNK is involved in MEK/ERK1/2 activation, we performed further co-immunoprecipitation experiments. These investigations indicated the interaction between phosphorylated JNK and phosphorylated MEK and ERK1/2. This binding was detected only in the presence of IL-1b. These observations suggest that JNK1 can be an upstream regulator for MEK/ERK1/2 signaling in IL-1β-induced COX-2 expression in feline synovial fibroblasts.

Previous studies investigated the cross-talk between JNK and MEK/ERK pathways. MEK/ERK activity has been reported to regulate JNK activity in apoptotic signaling in IEC-6 cells derived from rat intestine^50^. In a human astrocyte cell line (U-251), MEK phosphorylated JNK^51^. In *Xenopus laevis* oocytes injected with oncogenic (Val 12)-ras-p21, cross-talk between Raf/MEK/ERK and JNK pathways has been reported^51^. In these cells, the activation of MEK activated JNK in a positive feedback loop. However, here we demonstrated that MEK inhibitor had no effect on JNK activation in IL-1β-stimulated cells, suggesting that MEK fails to activate JNK. Therefore, it is unlikely that our system is a feedback loop. The existence of a negative cross-talk relationship between JNK and ERK signaling has also been demonstrated in COS-7 cells, in which sustained JNK activation inhibits ERK activation via uncoupling from MEK^52^. In Bcr/Abl+ human leukemia cells, the cooperation between inactivation of Raf/MEK/ERK pathway and activation of JNK pathway has been reported to be involved in histone deacetylase inhibitor-induced apoptosis^53^. However, since our results showed that JNK activation is necessary for MEK/ERK1/2 activation, IL-1β-induced cross-talk between JNK and MEK/ERK1/2 signaling in synovial fibroblasts is distinct from the negative cooperation. Therefore, it is most likely that JNK-regulated MEK/ERK1/2 signaling is a novel pathway.

We demonstrated that phosphorylated JNK binds to phosphorylated MEK/ERK1/2 in the IL-1β stimulation by the co-immunoprecipitation experiments. JNKs are a proline-directed serine/threonine kinase, and the recognition of JNK substrate needs the interaction of a defined JNK-binding motif of each protein substrate with the substrate-docking site on the C-terminal lobe of JNK^28^,^54^. Although various substrates of phosphorylated JNK have been reported^28^, the direct interaction between JNK and MEK/ERK1/2 remains unclear. Since JNK-induced c-Raf phosphorylation has been reported to result in MEK activation in human astrocyte cell line U-251^52^, it is likely that JNK activates MEK/ERK1/2 pathway via activation of protein kinases such as c-Raf.

We observed that the IL-1β-induced COX-2 mRNA expression was enhanced by JNK2 siRNA transfection (Fig. 7e). Therefore, we performed the experiments with JNK1 and JNK2 siRNAs together. In the cells transfected with JNK1 and JNK2 siRNAs, IL-1β-induced COX-2 mRNA expression was reduced compared with control (Fig. 7e). Taken together, it is likely that JNK1 contributes to COX-2 mRNA expression in the cells treated with IL-1β. However, the mechanism with the enhancement of IL-1β-induced COX-2 mRNA expression in the JNK2-knockdown cells is obscure. Although JNK2 appears to play as an inhibitory factor for JNK1 function in feline synovial fibroblasts, we need further studies.

**Figure 8:**
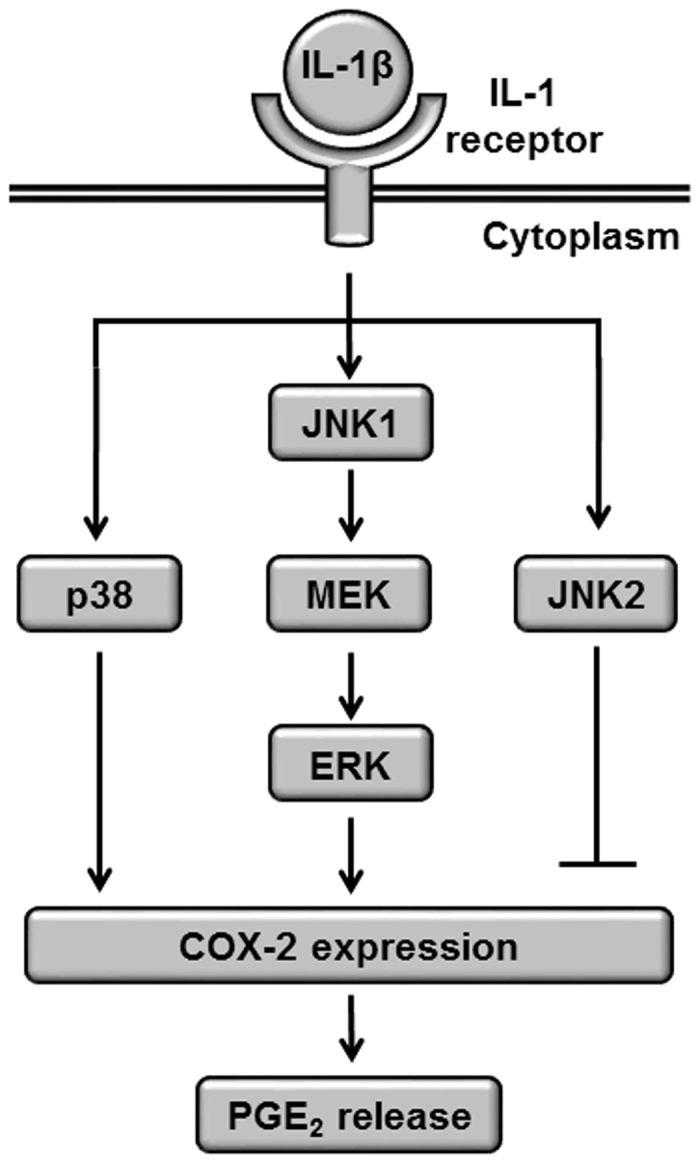
Schematic diagram of JNK1/MEK/ERK1/2 and p38 pathways in IL-1β-induced COX-2 expression in feline synovial fibroblasts. In IL-1β-stimulated fibroblasts, phosphorylation of JNK1 leads to the activation of MEK/ERK signaling, which contributes to COX-2 expression and prostaglandin E_2_ (PGE_2_) synthesis, whereas JNK2 pathway suppresses COX-2 expression. Activation of the p38 pathway also contributes to COX-2 expression and prostaglandin E_2_ (PGE_2_) synthesis independently of the JNK1/MEK/ERK1/2 pathway.

In conclusion, we demonstrated that JNK, MEK/ERK, and p38 MAPK signaling contribute to IL-1β-induced COX-2 expression and prostaglandin E_2_ release COX-2 in feline synovial fibroblasts. Moreover, we demonstrated that activation of the JNK isoform JNK1 is required to activate MEK/ERK1/2 signaling. A scheme consistent with the observations in IL-1β-induced feline synovial fibroblasts is provided in Fig. 8. Our observations indicate that JNK1/MEK/ERK1/2 signaling represent promising molecular targets for the development of therapeutic intervention in OA synovitis.

## Materials and Methods

### Materials

TRIzol and Lipofectamine 2000 were obtained from Life Technologies Co. (Carlsbad, CA). Thermal Cycler Dice Real Time System II, TP900 DiceRealTime v4.02B, SYBR Premix Ex Taq II, PrimeScript RT Master Mix, and CELLBANKER 1 plus medium were purchased from TaKaRa Bio Inc. (Shiga, Japan). Rabbit monoclonal antibodies against human total JNK-1 (t-JNK1, EPR140(2)), human total JNK-2 (t-JNK2, EP1595Y), human COX-1 (EPR5867), and rabbit polyclonal antibodies against COX-2 were obtained from Abcam (Cambridge, UK). Anti-total MEK (t-MEK, D1A5), anti-phospho-MEK (p-MEK), anti-rat total-ERK1/2 (t-ERK1/2, 137F5), anti-human phospho-ERK1/2 (p-ERK1/2, D13.14.4E), anti-human total-p38 (t-p38, D13E1), and anti-human phospho-p38 (p-p38, 3D7) rabbit monoclonal or polyclonal antibodies were obtained from Cell Signaling Technology Japan, K.K. (Tokyo, Japan). Rabbit polyclonal antibody against phospho-JNK was obtained from Promega, Co. (Madison, WI). MAPK inhibitors, SB239063, PD98059, FR180204, SP600125, and mouse monoclonal anti-β-actin antibody (AC74) were purchased from Sigma-Aldrich Inc. (St Louis, MO). ImageQuant LAS 4000 mini, horseradish peroxidase (HRP)-conjugated anti-mouse and anti-rabbit IgG antibodies, protein A plus G Sepharose, and ECL Western blotting Analysis System were obtained from GE Healthcare (Piscataway, NJ). Mini-PROTEAN TGX gel, polyvinylidene difluoride (PVDF) membranes, and iCycler, were purchased from Bio-Rad (Hercules, CA). Block Ace and Complete mini EDTA-free protease inhibitor mixture were obtained from Roche (Mannheim, Germany). Dulbecco’s modified Eagle’s medium (DMEM) with 1 g/L glucose was obtained from Wako Pure Chemical Industries, Ltd. (Osaka, Japan). An enzyme-linked immunosorbent assay (ELISA) kit for prostaglandin E_2_ was obtained from Cayman Chemical Co. (Ann Arbor, MI). StatMate IV was obtained from ATMS (Tokyo, Japan). A freezing vessel (BICELL) was obtained from Nihon Freezer Co., Ltd. (Tokyo, Japan).

### Cell culture

This study was approved by the Institutional Animal Care and Use Committee at the Kimura Animal Hospital (Approval numbers: KAH2014-001, KAH2015-001, KAH2015-002). All experiments were performed in accordance with the guidelines and regulations of Kimura Animal Hospital. Informed consent was obtained from all owners. Feline synovial membranes were obtained from healthy knee joints of domestic short-haired cats during reductive surgery of femoral fracture (n = 3, 4–7 years old, spayed female). The cats were premedicated with a subcutaneous injection of atropine sulfate (0.05 mg/kg; Mitsubishi Tanabe Pharma Co., Osaka, Japan.). Anesthesia was induced intravenously with propofol (4.0 mg/kg; Intervet K.K, Osaka, Japan) and maintained with 2.0% isoflurane (Intervet K.K.) and 100% oxygen was provided in an endotracheal tube. To minimize potential pain and infection, remifentanil hydrochloride (3 to 5 μg/kg/min; Janssen Pharmaceutical K.K, Tokyo, Japan) and cefazolin (22 mg/kg; Nichi-Iko Pharmaceutical Co., Ltd, Toyama, Japan) were administered intravenously before awakening.

Synovial membranes were collected from the lateral compartment of knee joints when surgical treatments for bone fracture were performed. Fibrous tissues were carefully removed. Feline synovial fibroblasts were isolated by explant culture using a previously described method^23^,^55^ with few modifications. Briefly, feline synovial membrane from the knee joint was collected and cut into 3-mm^2^ sections. Each explant was placed into a 90-mm petri dish. The attached explants were maintained in static culture in an incubator at 5% CO_2_ and 37 °C using DMEM with 1 g/L glucose supplemented with 10% fetal bovine serum. The medium was changed once a week, and after three weeks, feline synovial fibroblasts were obtained as outgrowth cells. The feline synovial fibroblasts were harvested using 0.25% trypsin-EDTA once they reached 90–95% confluence. After suspension, the cells were collected using CELLBANKER 1 plus medium at a density of 2 × 10^6^ cells/500 μL. The cell suspension was placed into a sterilized serum tube. The tubes were then put into a BICELL vessel and cryopreserved at −80 °C. The serum tubes were removed from the BICELL vessel and immersed into a 37 °C water bath. Before the experiments, the thawed-out cell suspension was transferred to a centrifuge tube contained DMEM with 1 g/L glucose containing 10% fetal bovine serum and centrifuged at 300 *g* for 3 min. The supernatant was removed and the pellet was suspended in DMEM with 1 g/L glucose containing 10% fetal bovine serum and transferred to a 75-cm^2^ culture flask. Static cultures were then maintained under the same conditions as those before the cryopreservation. Cells were harvested using 0.25% trypsin-EDTA once they reached approximately 90% confluence. Then, the collected cells were seeded at a density of 1 × 10^6^ cells per 75-cm^2^ culture flask. The feline synovial fibroblasts used for all experiments were between passages six to eight. For each experiment, cells from three different animals were used.

### RT-PCR

Total RNA was extracted from feline synovial fibroblasts using TRIzol reagent according to the manufacturer’s instructions. Total RNA concentration was measured spectrophotometrically by reading absorbance at 260/280 nm. First-strand cDNA was generated using 500 ng of total RNA, using the PrimeScript RT Master Mix. PCRs were performed using 2 μL of first-strand cDNA, Ex Taq polymerase, and primers specific for feline β-actin, JNK1, JNK2, and JNK3 (see Supplementary Table S1) in a total reaction volume of 10 μl. PCRs were conducted using iCycler. The thermal cycler was programmed for initial denaturation at 94 °C for 2 min, followed by 30 cycles of denaturation at 94 °C for 30 sec, primer annealing at 55 °C for 30 sec, and primer extension at 72 °C for 30 sec. The PCR products were separated using 2% agarose gel electrophoresis, followed by ethidium bromide staining and visualization under UV light. mRNA expression levels in each sample were normalized to that of β-actin.

### Real-time RT-PCR

Total RNA was extracted from feline synovial fibroblasts using TRIzol reagent. First-strand cDNA synthesis was performed using 500 ng of total RNA, using the PrimeScript RT Master Mix. Real-time RT-PCR was carried out using 2 μL of the first-strand cDNA, SYBR Premix Ex Taq II, and primers specific for feline COX-1, COX-2, JNK1, JNK2, and β-actin (a housekeeping protein used as internal control) (see Supplementary Table S1) in a total reaction volume of 25 μL. Real-time RT-PCR of no-template controls was carried out using 2 μL of RNase- and DNA-free water. Additionally, real-time PCR of no-reverse transcription control was carried out using 2 μL of each RNA sample. PCR was performed using Thermal Cycler Dice Real Time System II with the following protocol: 1 cycle of denaturation at 95 °C for 30 sec, 40 cycles of denaturation at 95 °C for 5 sec, and annealing/extension at 60 °C for 30 sec. The analyses of results were performed by the second derivative maximum method and the comparative cycle threshold (ΔΔ Ct) method using real-time RT-PCR analysis software. The amplification of β-actin from the same amount of cDNA was applied as an endogenous control, while cDNA amplification from feline synovial fibroblasts at time 0 was used as the calibration standard.

### Western blotting

To collect the protein samples, cells were lysed with a lysis buffer containing 20 mM HEPES, 1 mM PMSF, 10 mM sodium fluoride, and a complete mini EDTA-free protease inhibitor cocktail at pH 7.4. The protein concentrations were adjusted using the Bradford method^56^. Extracted proteins were boiled at 95 °C for 5 min in SDS buffer, were loaded into separate lanes of 7.5% or 12% Mini-PROTEAN TGX gel, and electrophoretically separated. Separated proteins were transferred to PVDF membranes. The membranes were treated with Block Ace for 50 min at room temperature, and incubated with primary antibodies [COX-2 (1:1,000), COX-1 (1:100), p-MEK (1:1,000), t-MEK (1:1,000), p-ERK1/2 (1:1,000), t-ERK1/2 (1:1,000), p-p38 (1:1,000), t-p38 (1:1,000), p-JNK (1:1,000), t-JNK (1:1,000), and β-actin (1:10,000)] for 120 min at room temperature. After washing, the membranes were treated with an HRP-conjugated anti-rabbit or anti-mouse IgG antibody (1:10,000) for 90 min at room temperature. ECL Western Blotting Analysis System was used for detection of immunoreactivity. Chemiluminescent signals of the membranes were detected using ImageQuant LAS 4000 mini.

### Prostaglandin E_2_ assay

Feline synovial fibroblasts were seeded at a density of 3.0 × 10^5^ cells per well in 6-well culture plates. These cells were stimulated with feline recombinant IL-1β after starvation for 24 h, and culture supernatants were collected. To measure prostaglandin E_2_ concentrations in the culture supernatant, an ELISA kit was used according to the manufacturer’s instructions.

### Immunoprecipitation

Total cell lysates (100 μg) was precleared with protein A plus G Sepharose before incubation with specific antibodies, followed by addition of protein A plus G Sepharose. The total cell lysate was incubated with 5 μg anti-p-MEK, p-ERK and p-JNK antibody at 4 °C for 18 h. The precipitated proteins were dissolved in SDS buffer and boiled at 95 °C for 5 min before electrophoresis. Finally, the precipitated proteins were analyzed by western blotting.

### siRNA transfection

Feline synovial fibroblasts, seeded at a density of 1 × 10^5^ cells/35 mm dish or 5 × 10^5^ cells/90 mm dish, were transfected using Opti-MEM containing 5 μl/ml Lipofectamine 2000 and 50 nM JNK-1, JNK-2, or scramble siRNA for 6 h. Supplementary Table S2 shows sequences of siRNA. After that, siRNA efficiency was tested by real-time RT-PCR using primers specific for feline JNK-1 or JNK-2, and western blotting using an anti-JNK-1 antibody (1:1,000) or an anti-JNK-2 antibody (1:1,000).

### Statistical analysis

The data from all experiments are presented as the mean ± standard error of measurement. Statistical analyses were performed using StatMate IV. The data from the immunoprecipitation study and the data from the time-course study were analyzed using two-tailed Student’s t-test and two-way analysis of variance (ANOVA), respectively. The data from other experiments were analyzed using one-way ANOVA. Tukey’s test was used as post-hoc analysis. *P*-values less than 0.05 were considered statistically significant.

## Additional Information

**How to cite this article**: Kitanaka, T. *et al*. JNK activation is essential for activation of MEK/ERK signaling in IL-1β-induced COX-2 expression in synovial fibroblasts. *Sci. Rep.* **7**, 39914; doi: 10.1038/srep39914 (2017).

**Publisher's note:** Springer Nature remains neutral with regard to jurisdictional claims in published maps and institutional affiliations.

## Supplementary Material

Supplementary Information
