# Supplementary material for: JNK activation is essential for activation of MEK/ERK signaling in IL-1β-induced COX-2 expression in synovial fibroblasts
Source: Sci Rep. 2017 Jan 5;7:39914. doi: 10.1038/srep39914 (PMC5215076; doi:10.1038/srep39914)
Supplement: Supplementary Information [file srep39914-s1.pdf]

Title: JNK activation is essential for activation of MEK/ERK signaling in IL-1β-induced COX-2 expression in synovial fibroblasts

Author list: Taku Kitanaka [1,a], Rei Nakano [1,a], Nanako Kitanaka [1,] Taro Kimura [2], Ken Okabayashi [1], Takanori Narita [1] & Hiroshi Sugiya [1,] *

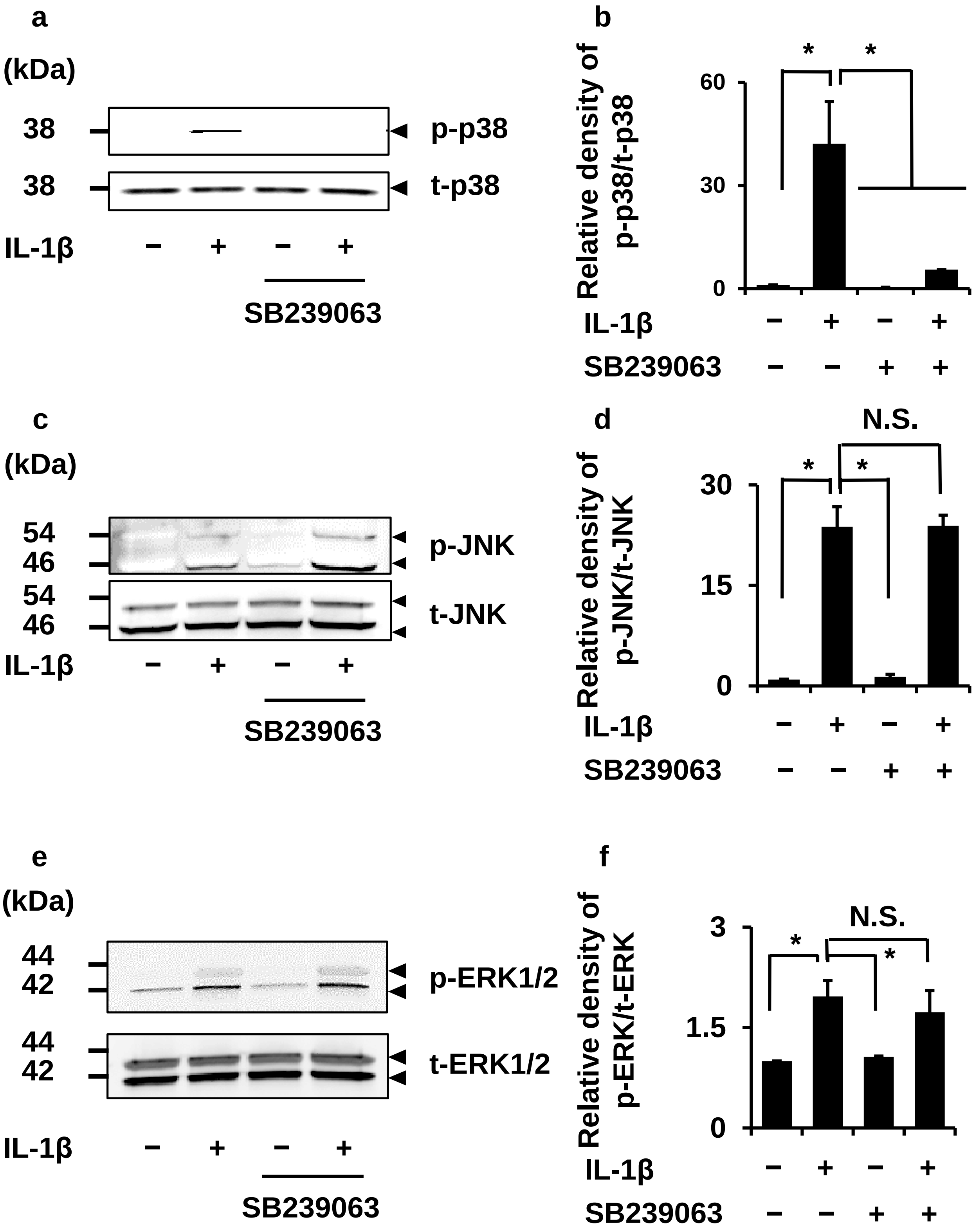

Fig. S1 The effect of the p38 inhibitor on the IL-1β-induced phosphorylation of p38, JNK and ERK1/2. When the cells were pretreated with the p38 inhibitor SB239063 (20 μM), the p38 inhibitor clearly attenuated the feline recombinant IL-1β-induced p38 phosphorylation (**a**). The p38 inhibitor had no effect on the IL-1β-induced phosphorylation of JNK (**c**) and ERK1/2 (**e**). Relative density of p-p38 (**b**), p-JNK (**d**) and p-ERK (**f**) compared with that of the absent of IL-1β is described. Results are presented as mean ± SE from 3 independent experiments. *$P < 0.05$.

Figure S1a. first row original figure

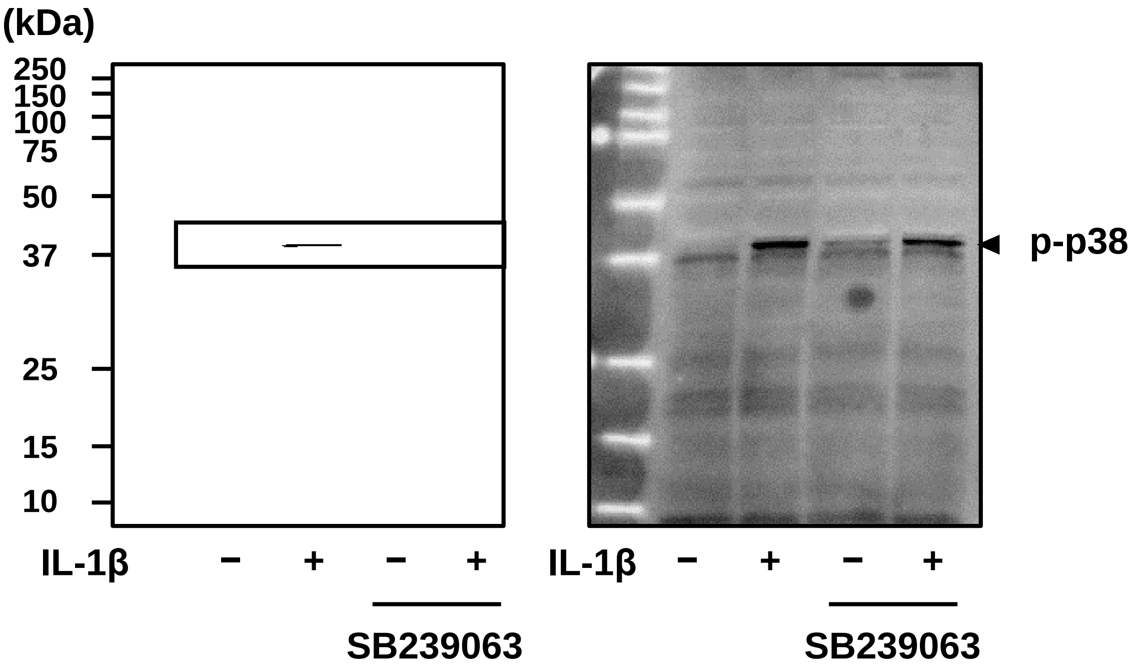

Figure S1a. second row original figure

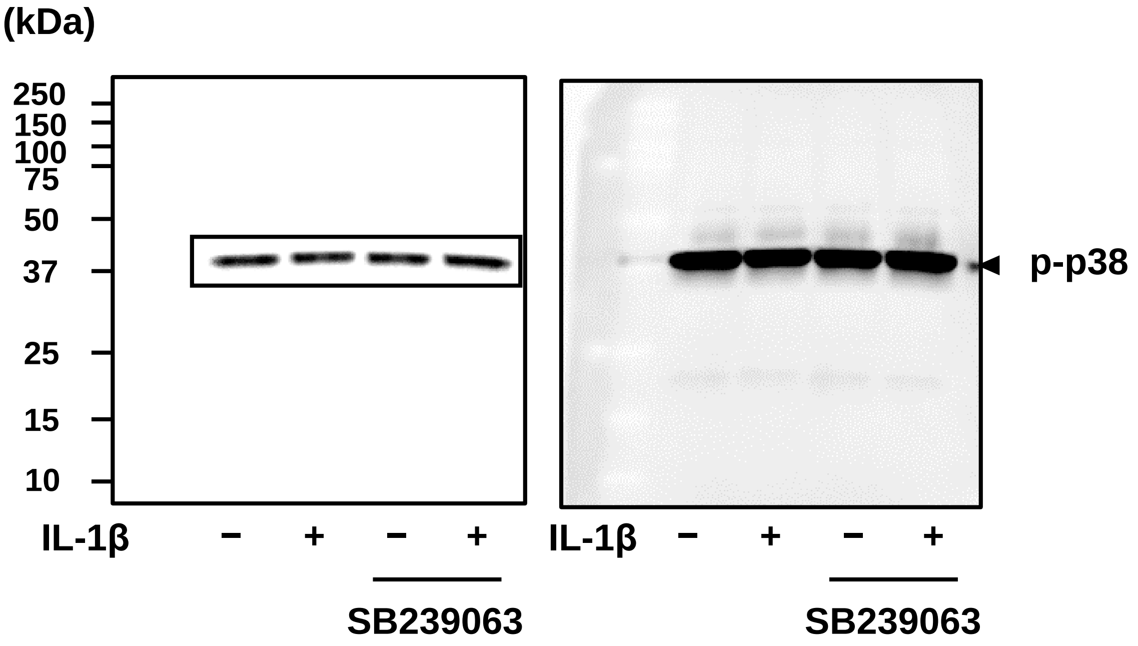

Fig. S1 (continue) Uncropped images for the blots shown in Figure S1a.

Figure S1c. first row original figure

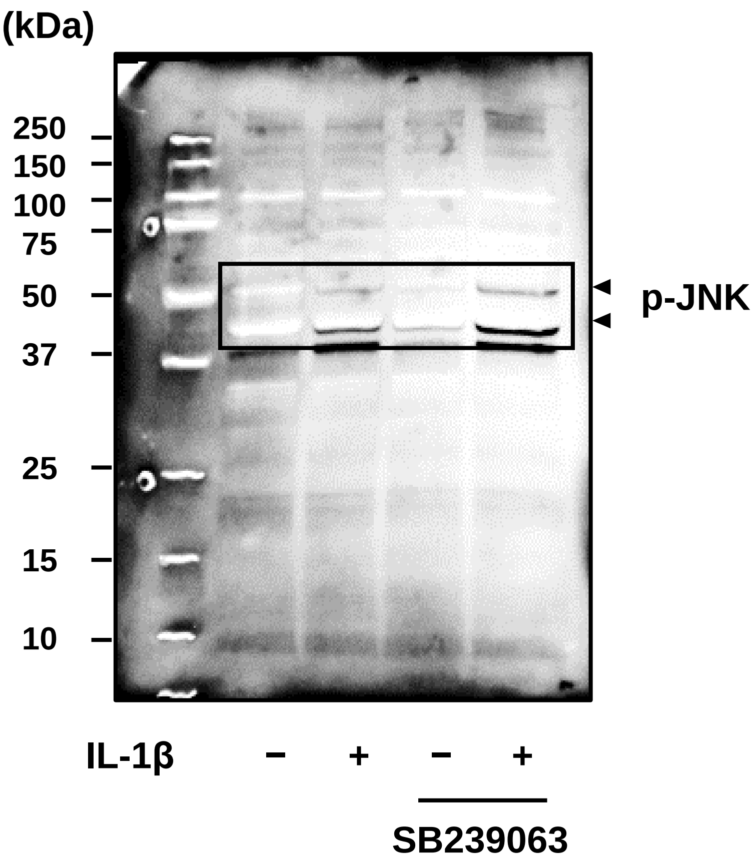

Figure S1c. second row original figure

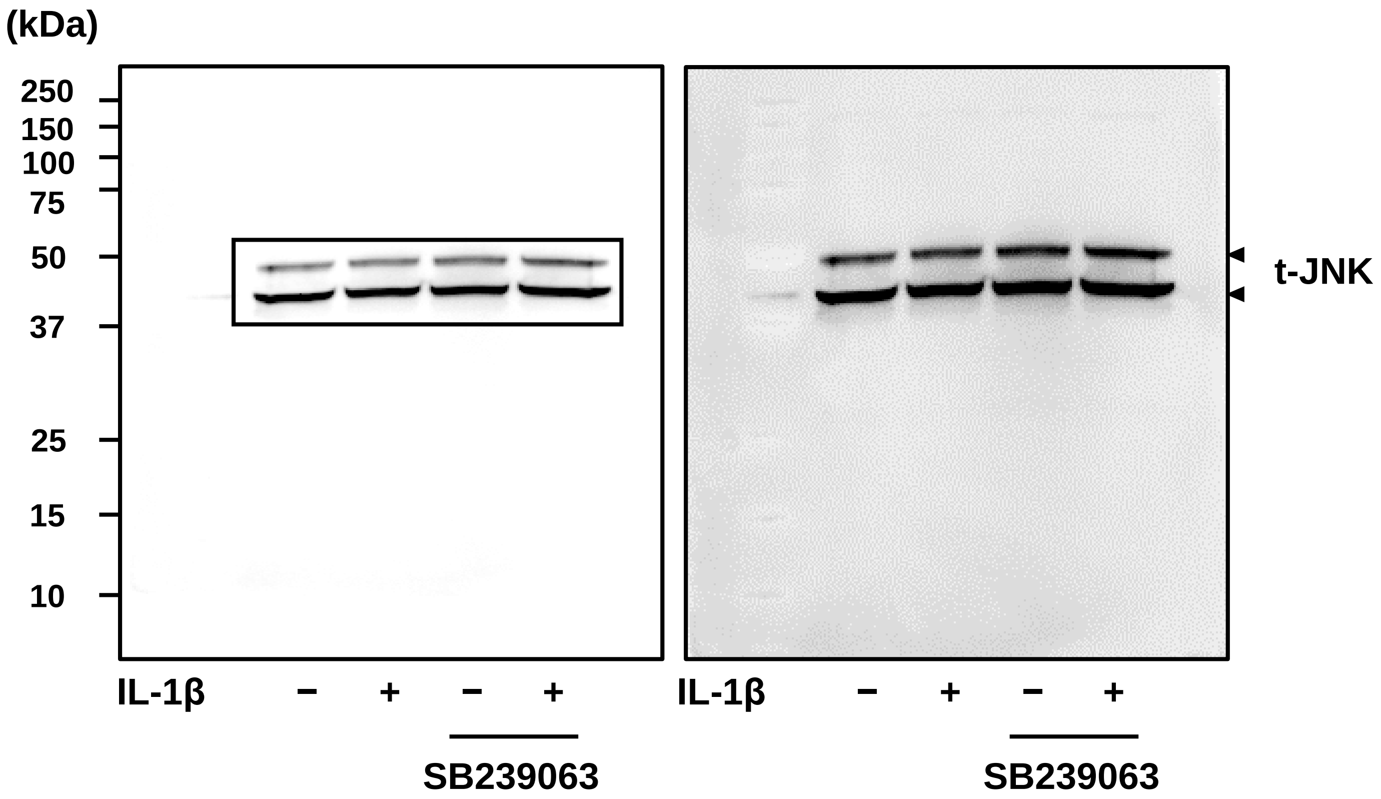

Fig. S1 (continue) Uncropped images for the blots shown in Figure S1c.

Figure S1e. first row original figure

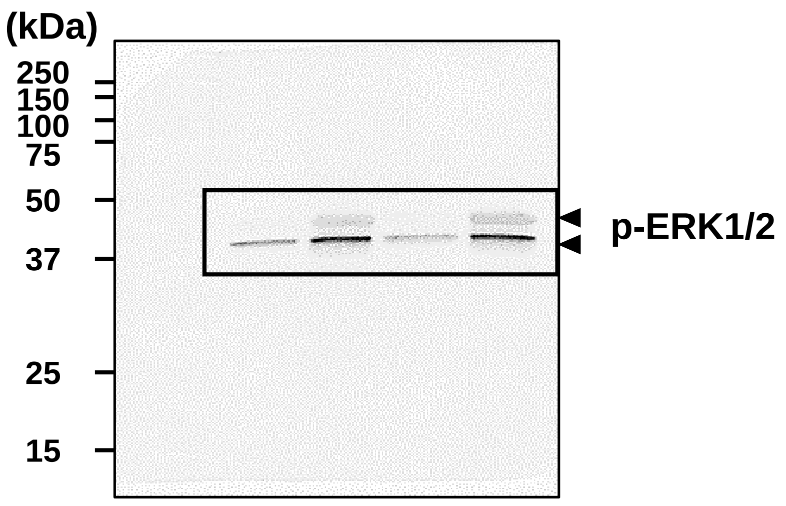

Figure S1e. second row original figure

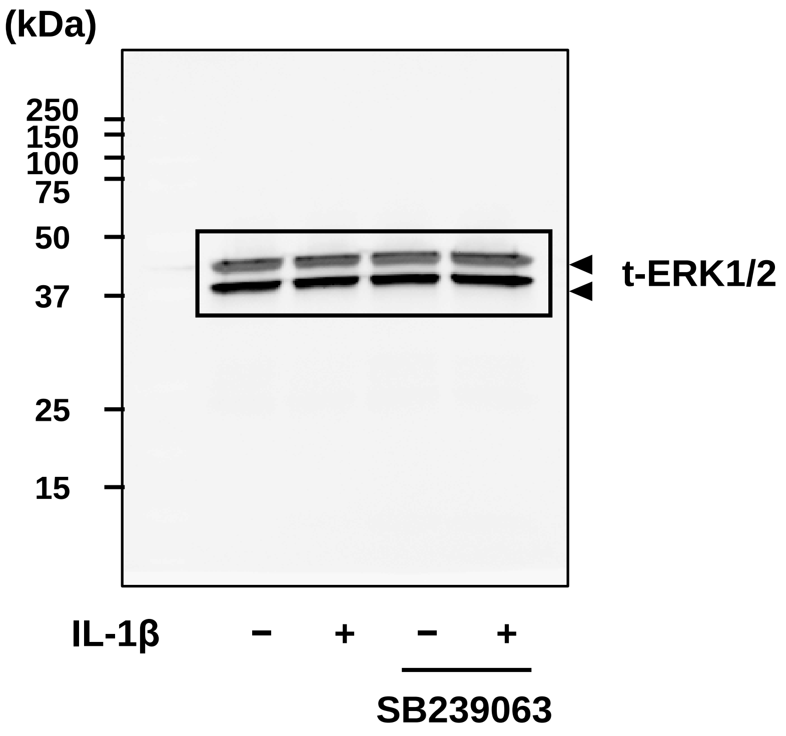

Fig. S1 (continue) Uncropped images for the blots shown in Figure S1e.

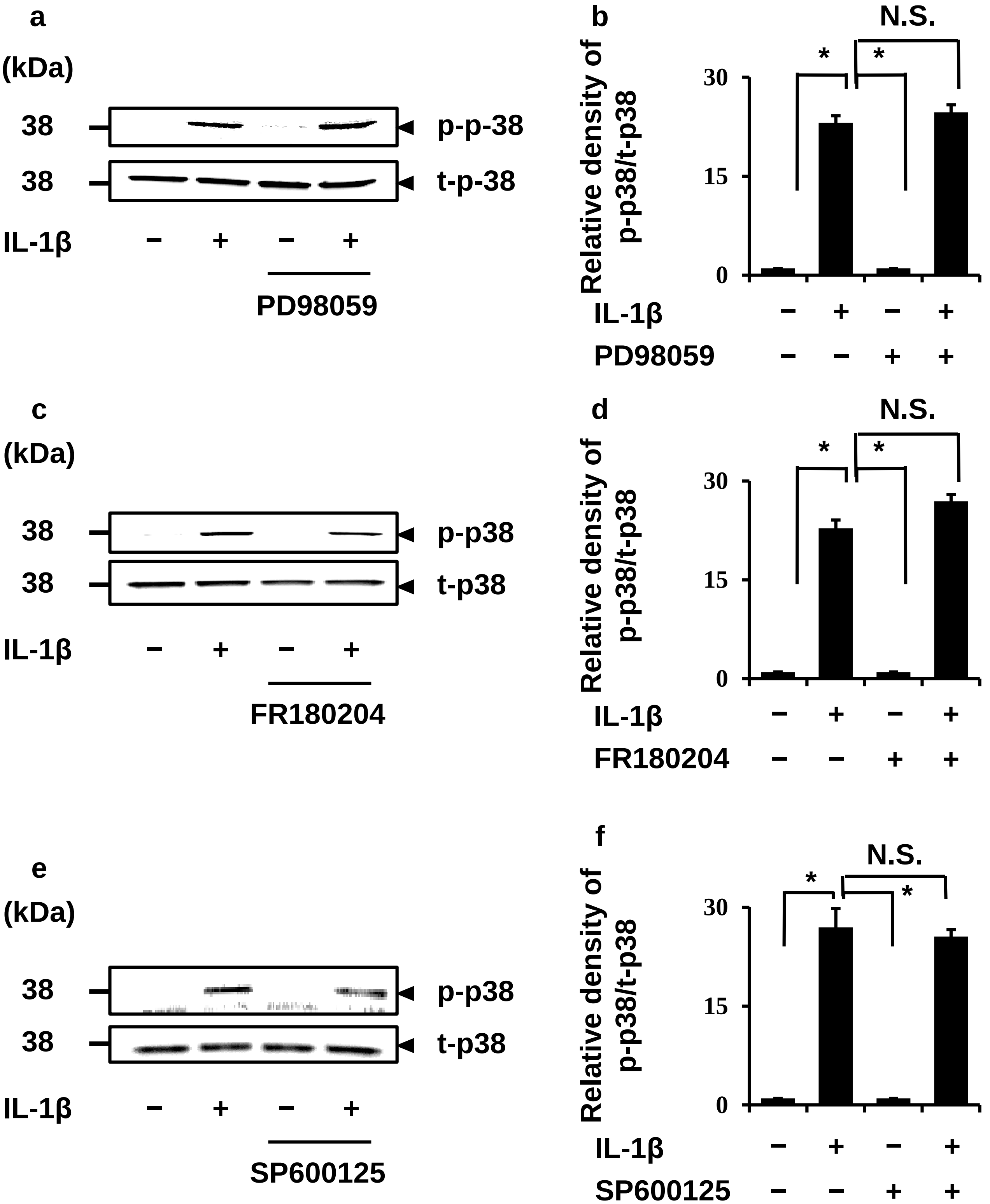

Fig. S2 The effect of the MEK, ERK or JNK inhibitor on the IL-1β-induced phosphorylation of p38. A. The MEK inhibitor PD98059 (a; 50 μM), the ERK inhibitor FR180204 (c; 50 μM) or the JNK inhibitor SP600125 (e; 10 μM) had no effect on the IL-1β-induced phosphorylation of p38. Relative density of p-p38 (**b**, **d**, **f**) compared with that of the absent of IL-1β is described. Results are presented as mean ± SE from 3 independent experiments. **P* < 0.05.

Figure S2a. first row original figure

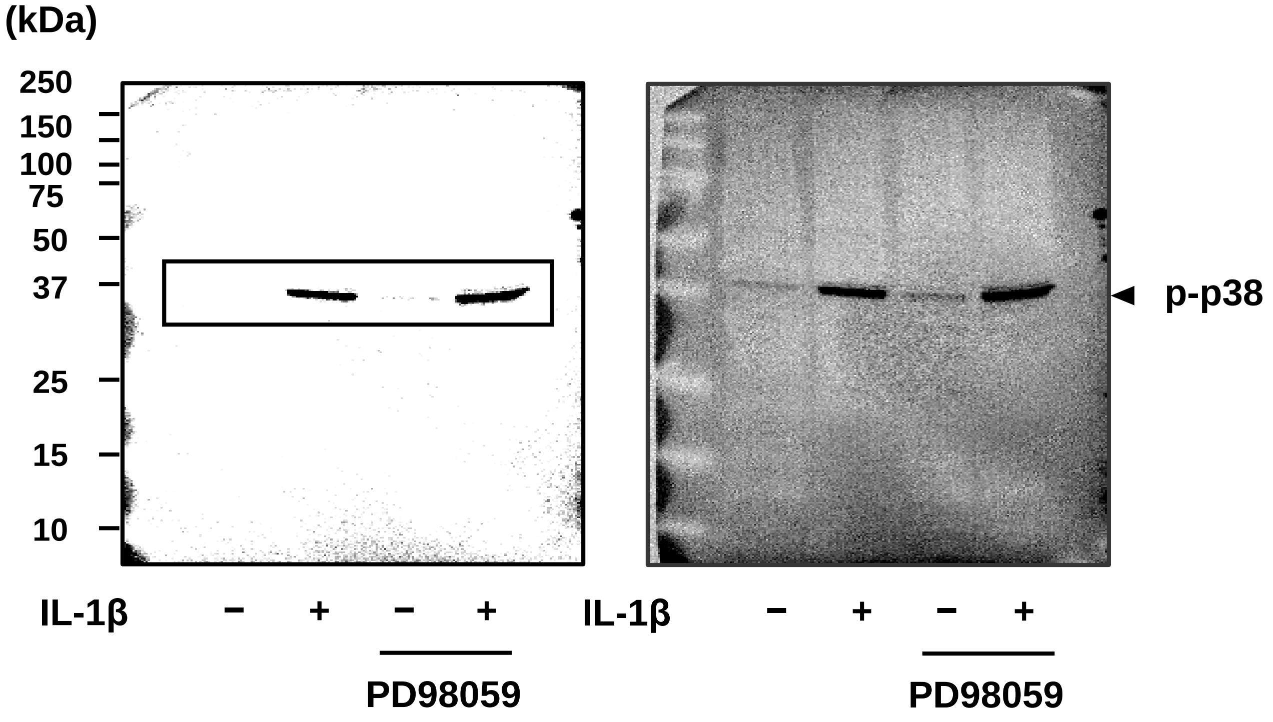

Figure S2a. second row original figure

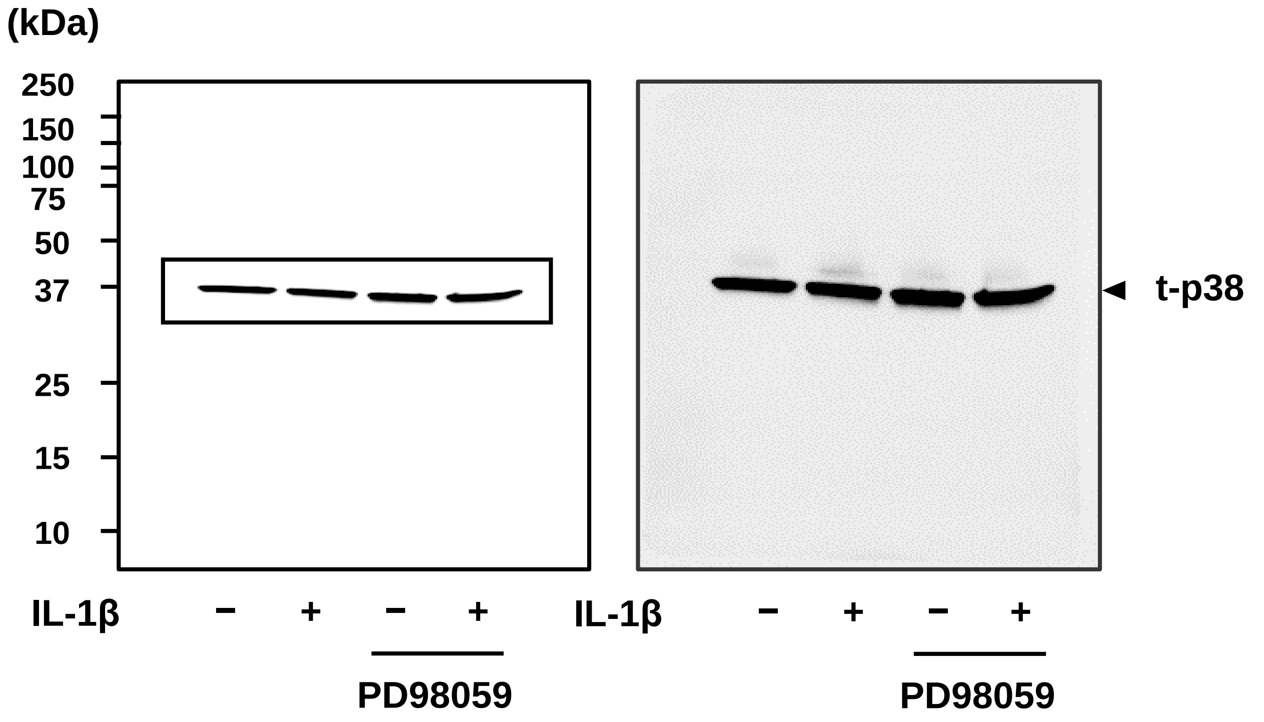

Fig. S2 (continue) Uncropped images for the blots shown in Figure S2a.

Figure S2c. first row original figure

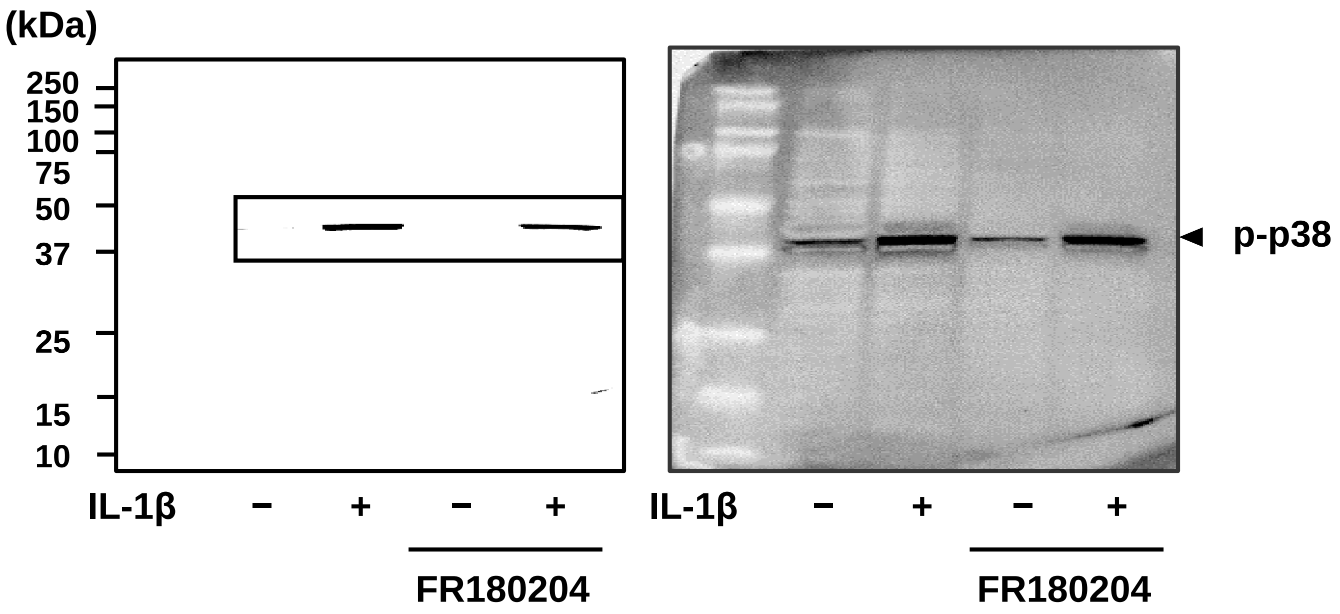

Figure S2c. second row original figure

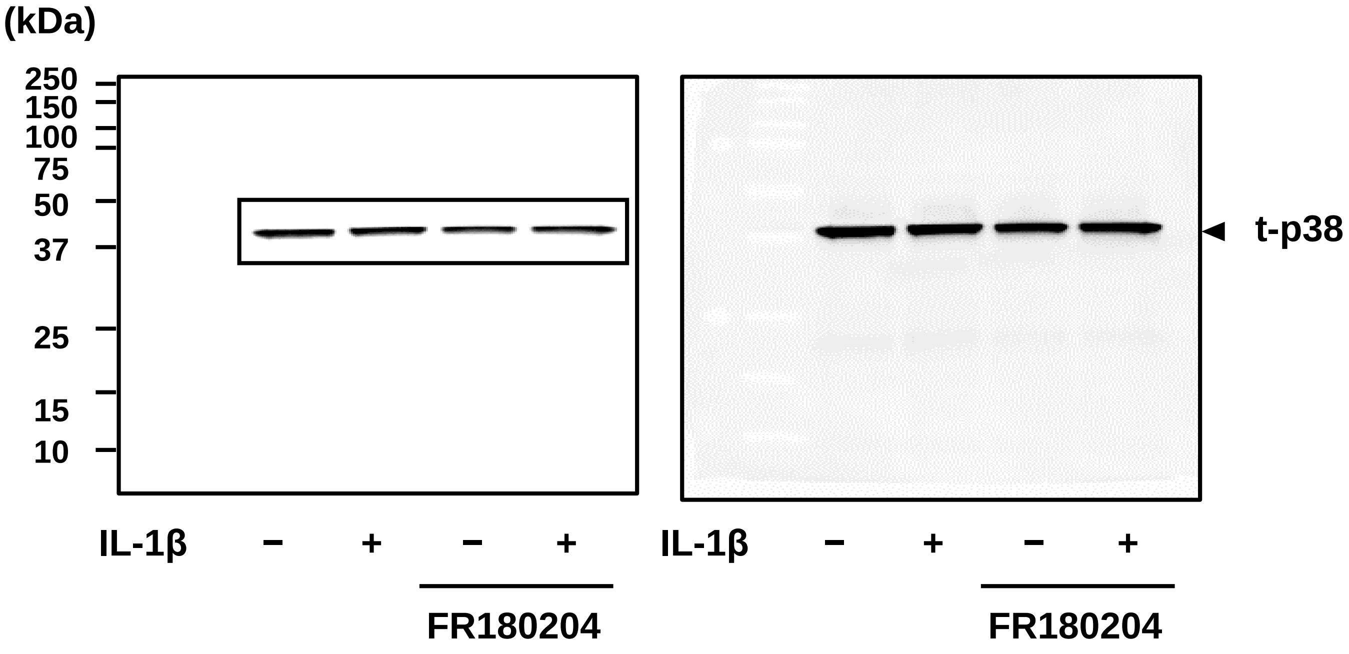

Fig. S2 (continue) Uncropped images for the blots shown in Figure S2c.

Figure S2e. first row original figure

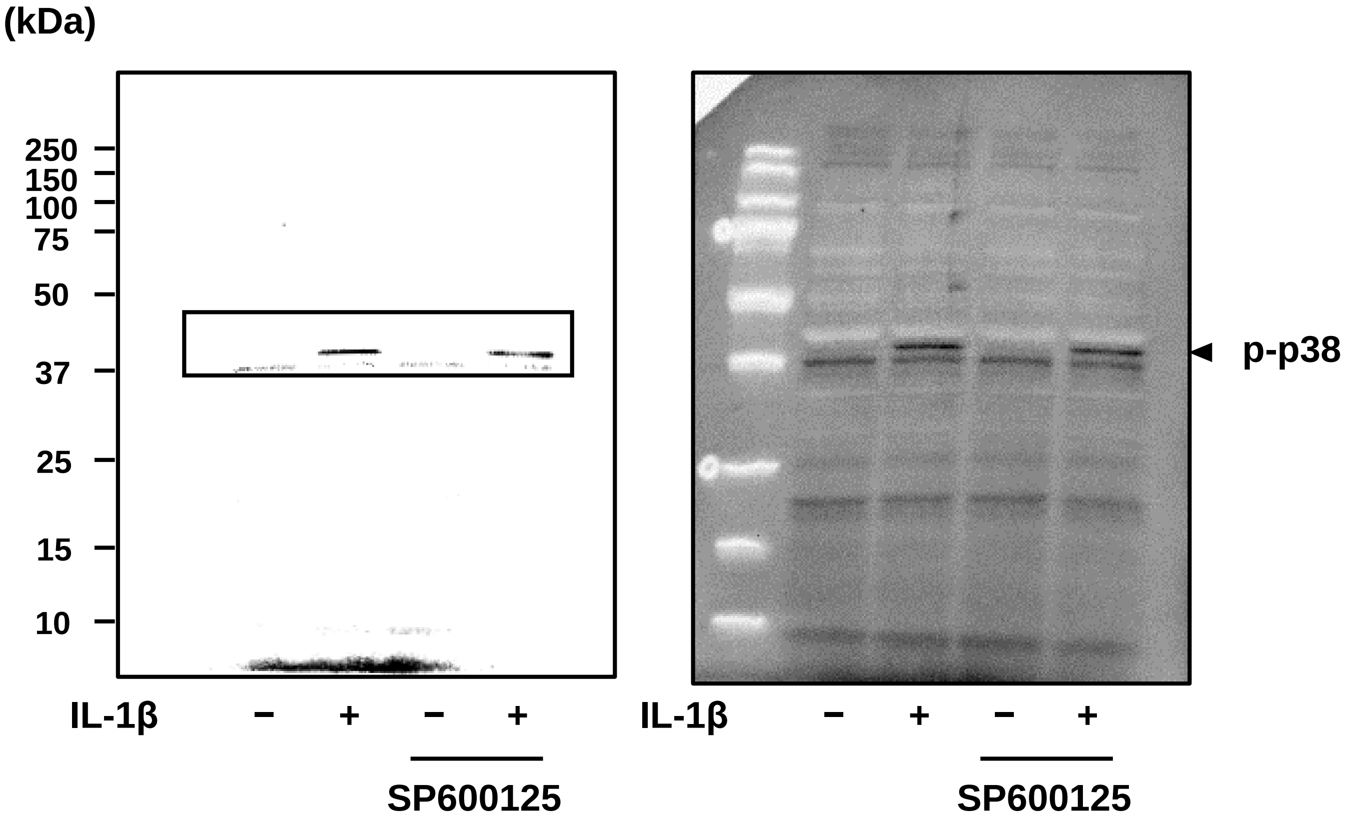

Figure S2e. second row original figure

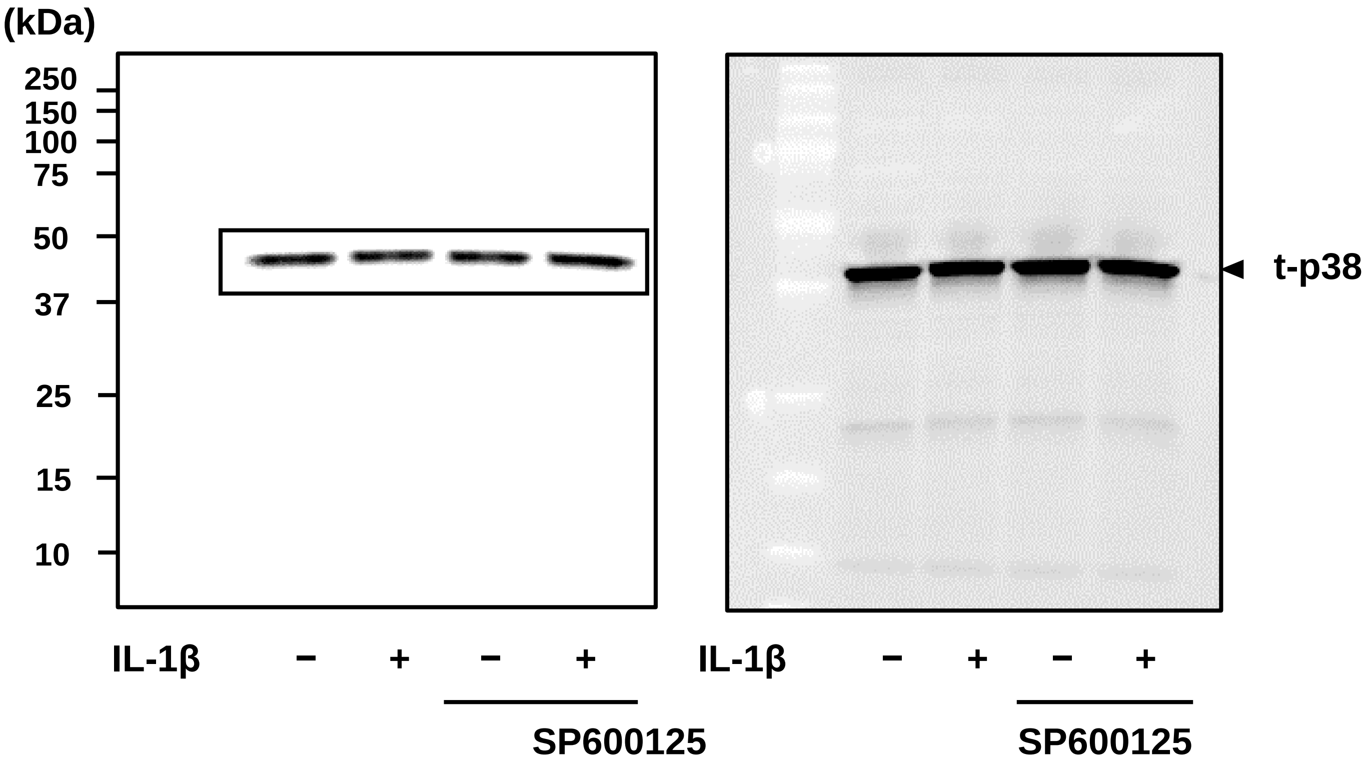

Fig. S2 (continue) Uncropped images for the blots shown in Figure S2e.

Figure 1f. first row original figure

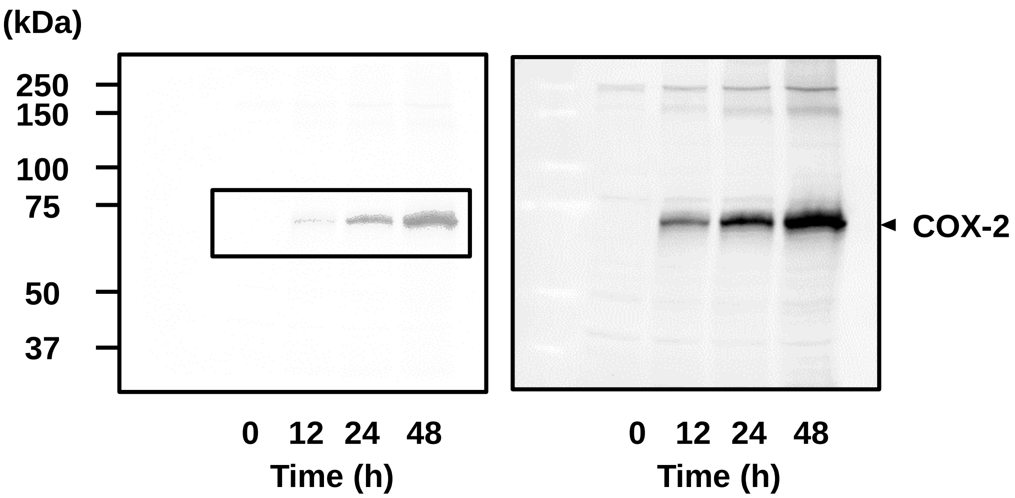

Figure 1f. second row original figure

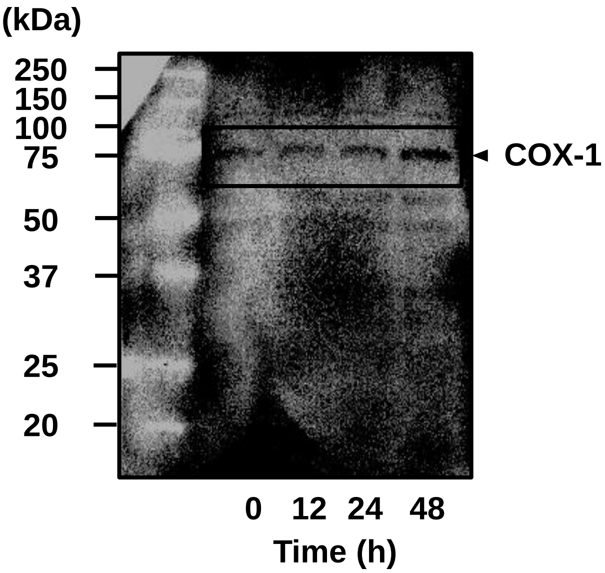

Figure 1f. third row original figure

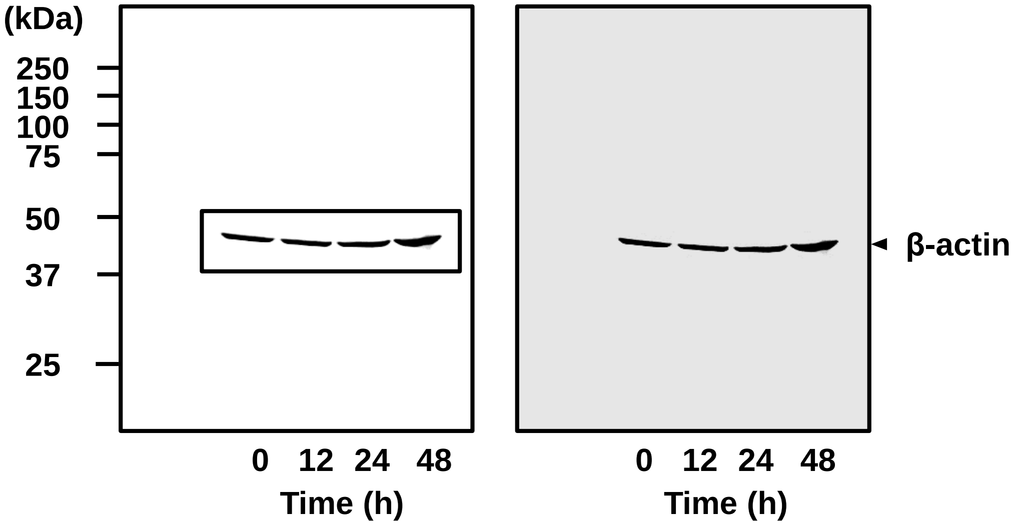

Fig. S3 Uncropped images for the blots shown in Figure 1f.

Figure 3a. first row original figure

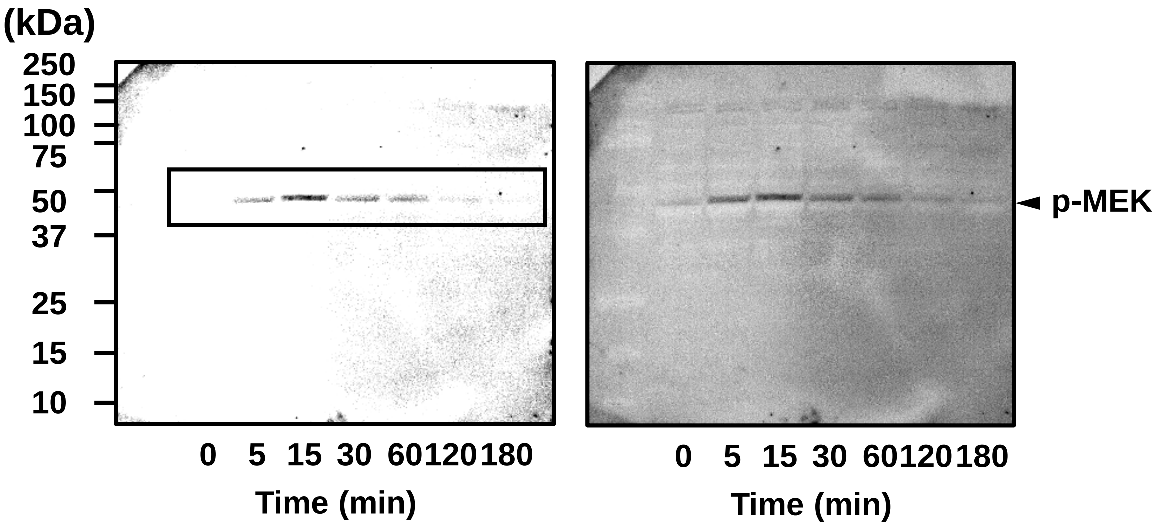

Figure 3a. second row original figure

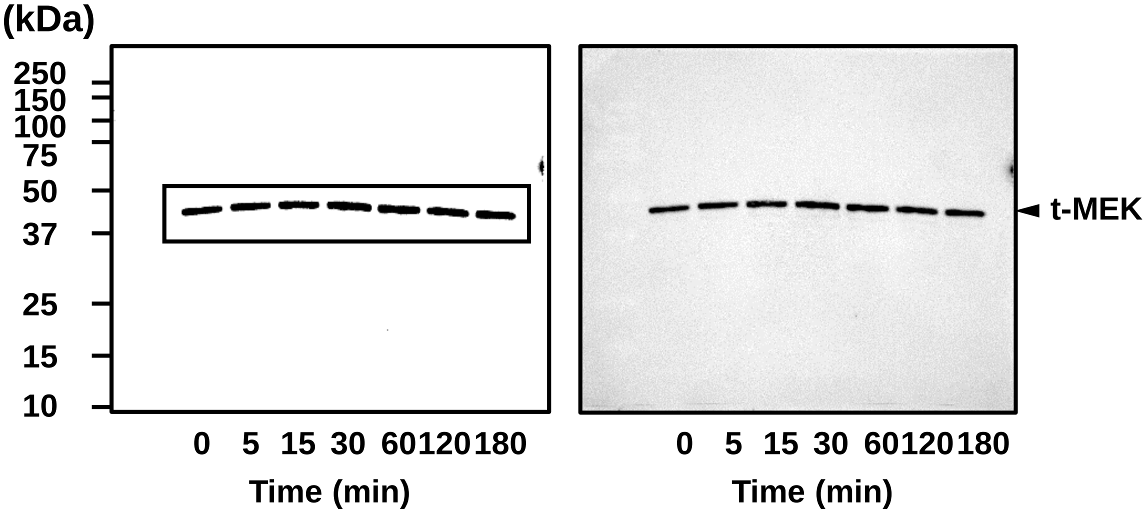

Fig. S4 Uncropped images for the blots shown in Figure 3a

Figure 3a. third row original figure

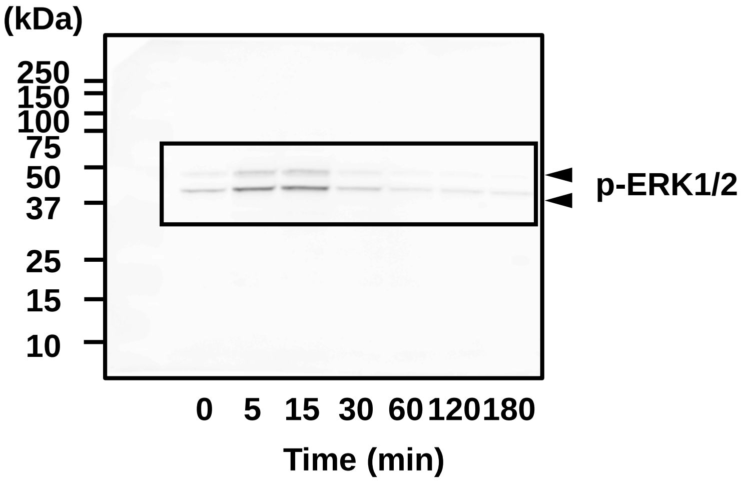

Figure 3a. fourth row original figure

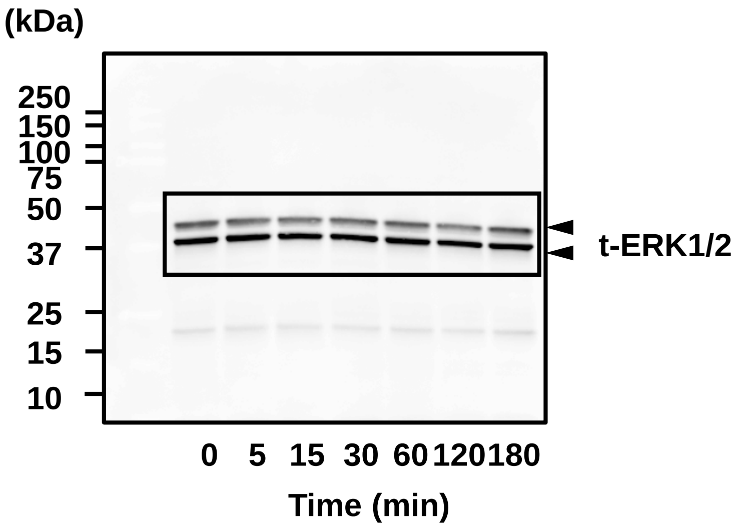

Fig. S4 (continue) Uncropped images for the blots shown in Figure 3a.

Figure 3a. fifth row original figure

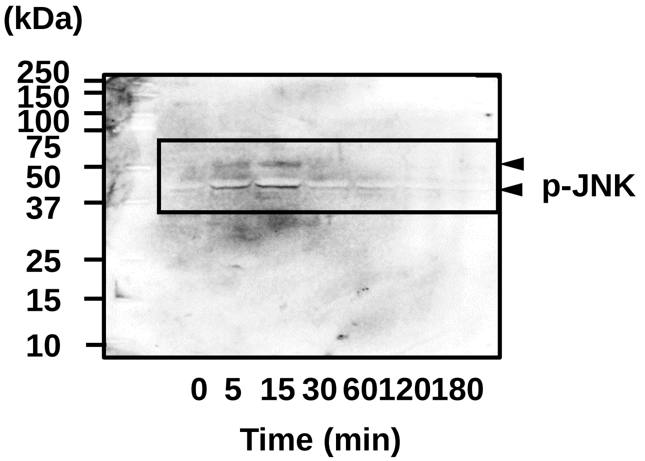

Figure 3a. sixth row original figure

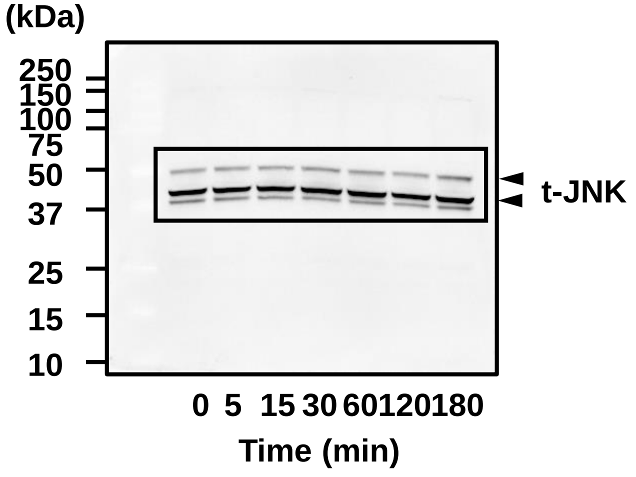

Fig. S4 (continue) Uncropped images for the blots shown in Figure 3a.

Figure 3a. seventh row original figure

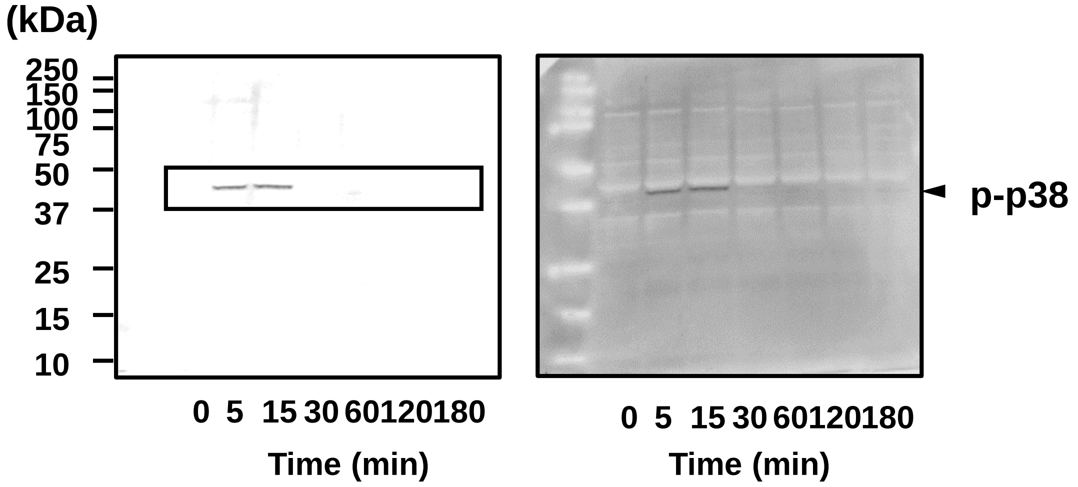

Figure 3a. eighth row original figure

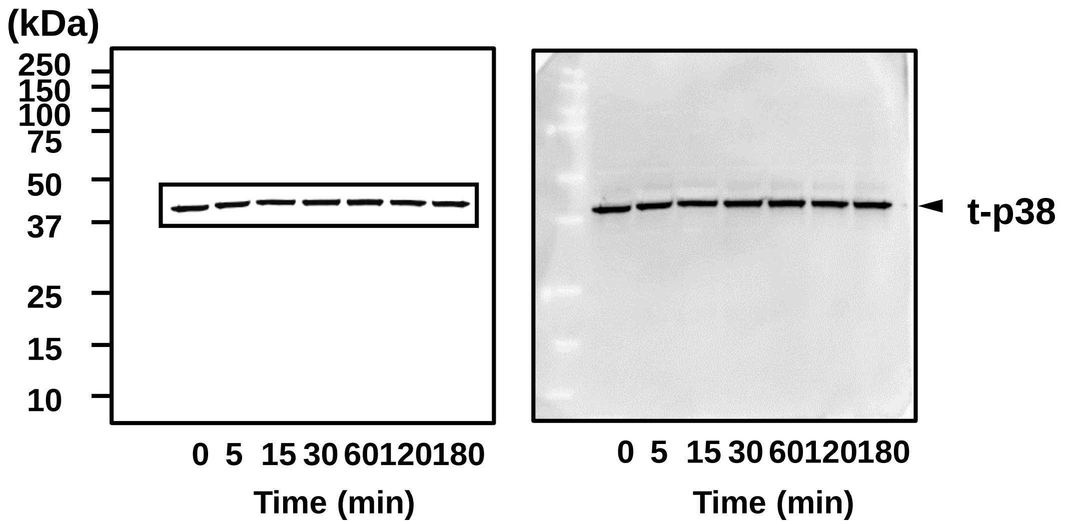

Fig. S4 (continue) Uncropped images for the blots shown in Figure 3a.

Figure 4a. original figure

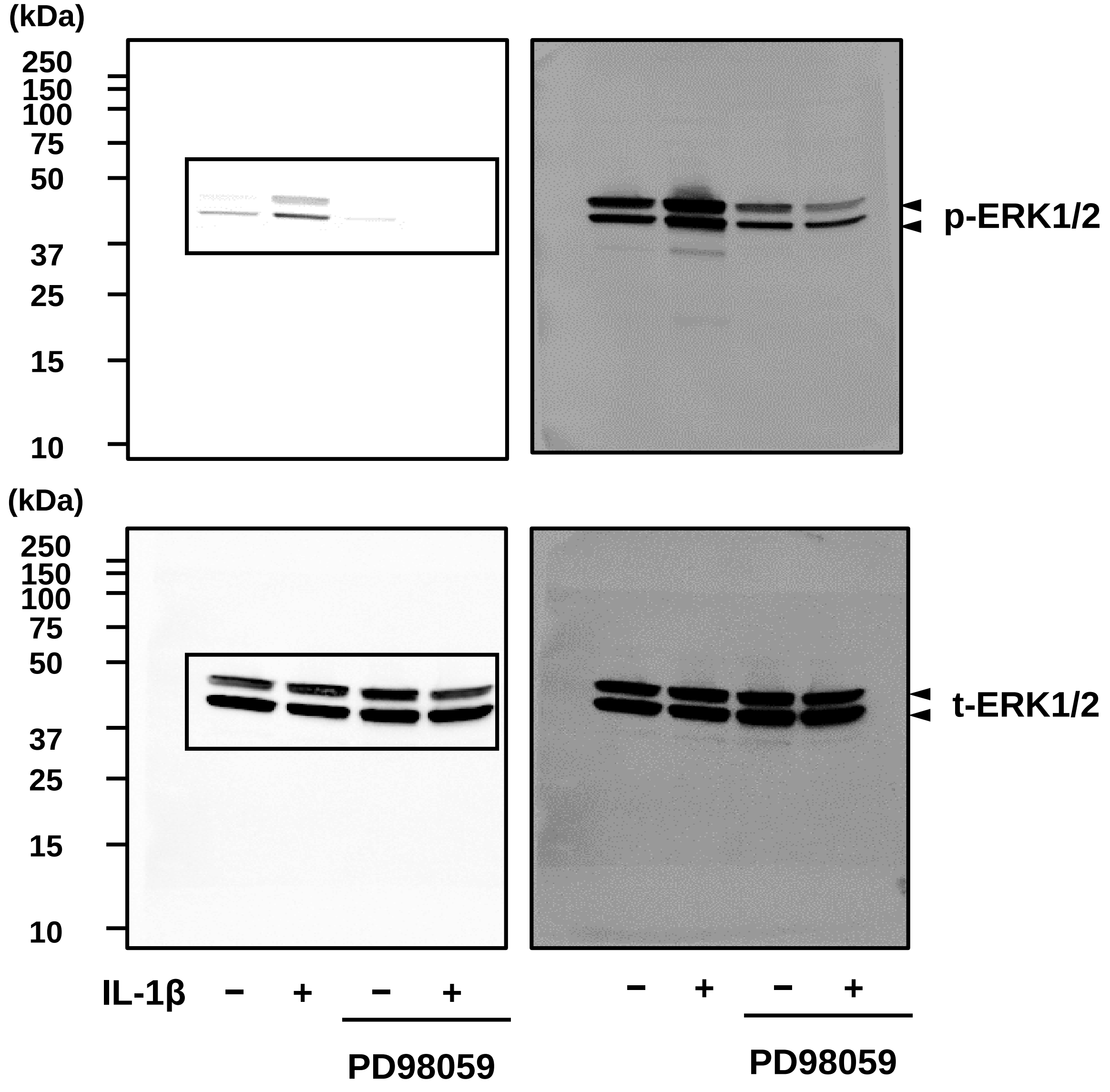

Fig. S5 Uncropped images for the blots shown in Figure 4a.

Figure 4c. original figure

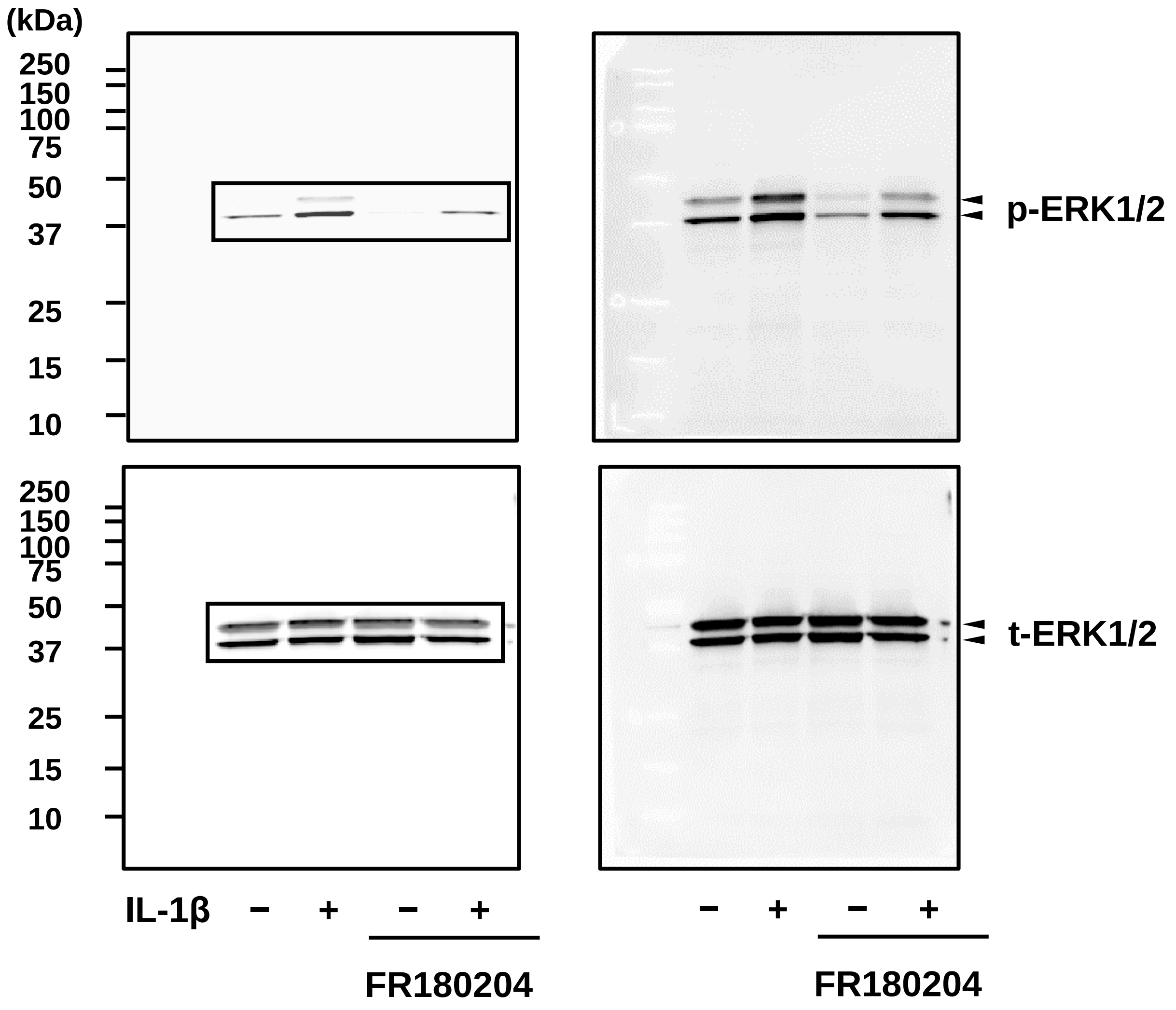

Fig. S6 Uncropped images for the blots shown in Figure 4c.

Figure 4e. original figure

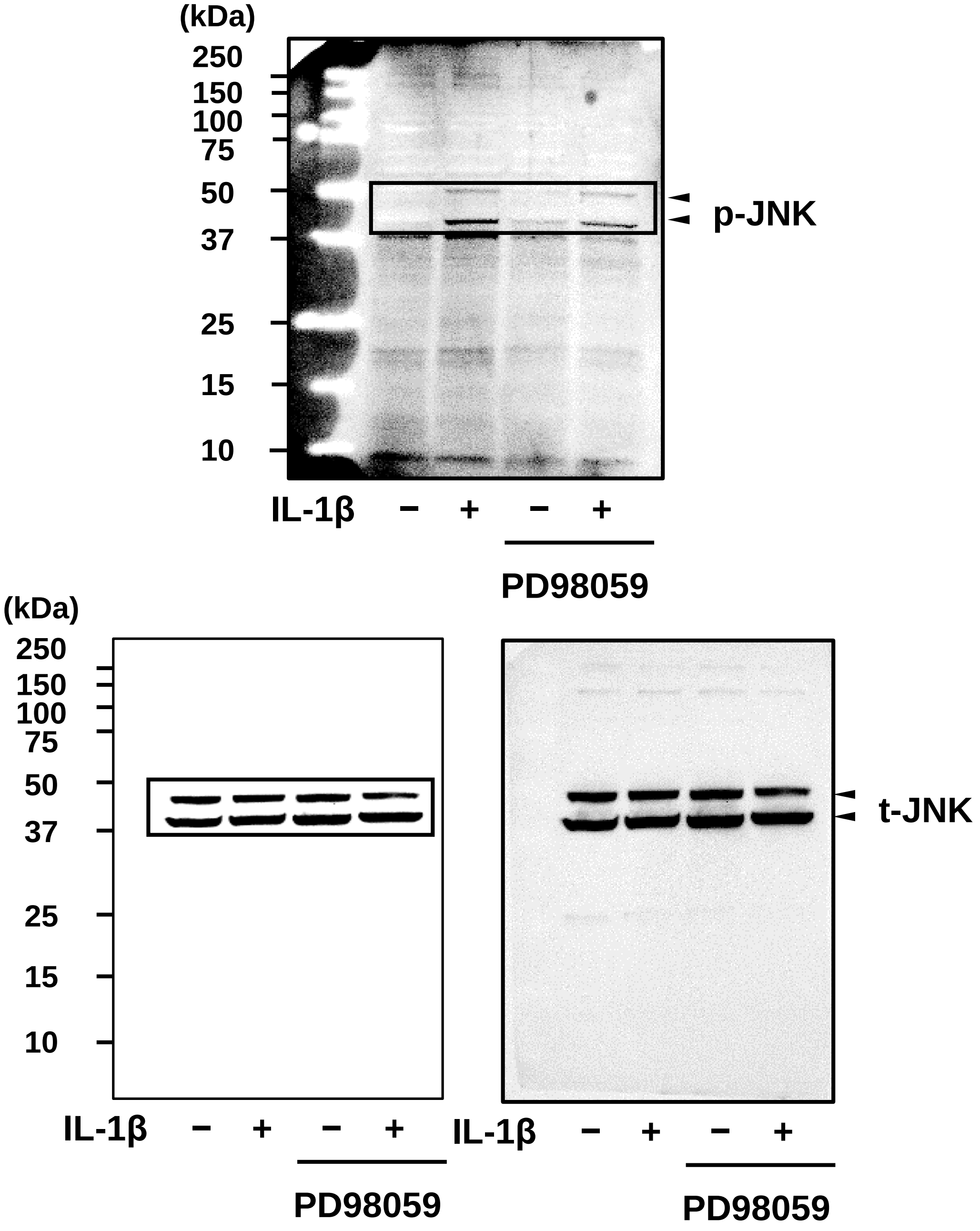

Fig. S7 Uncropped images for the blots shown in Figure 4e.

Figure 4g. original figure

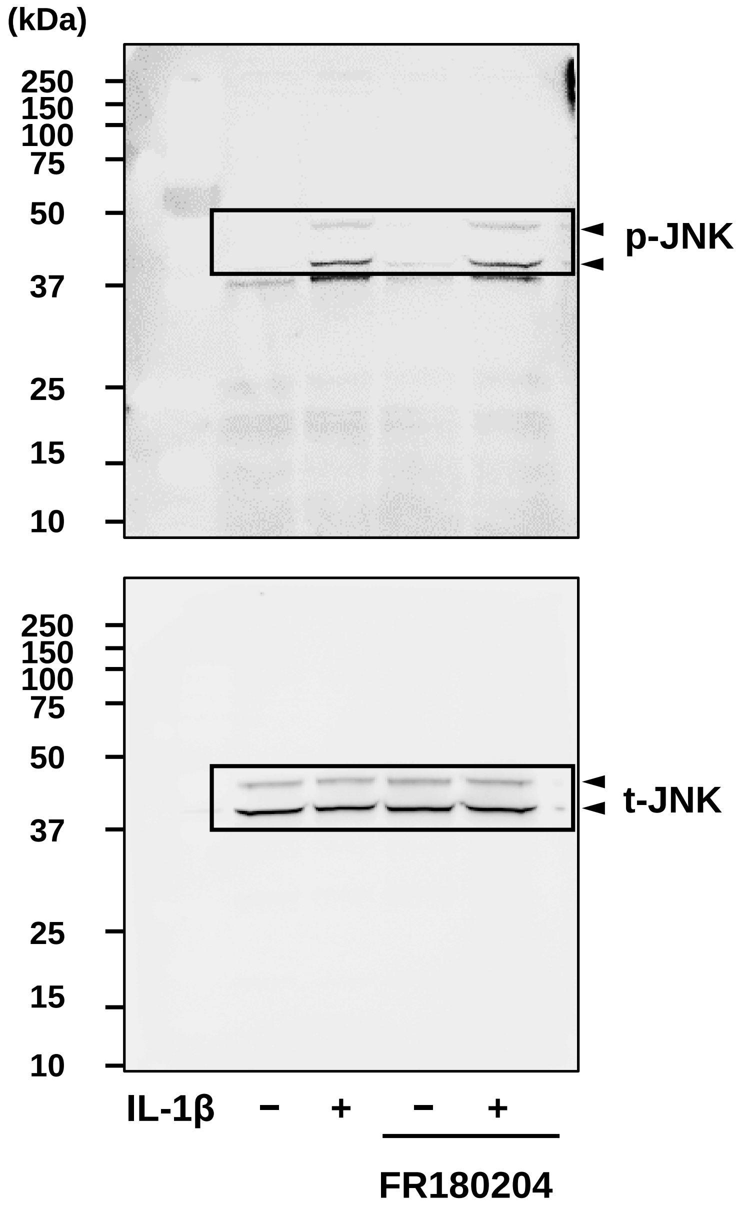

Fig. S8 Uncropped images for the blots shown in Figure 4g.

Figure 5a. original figure

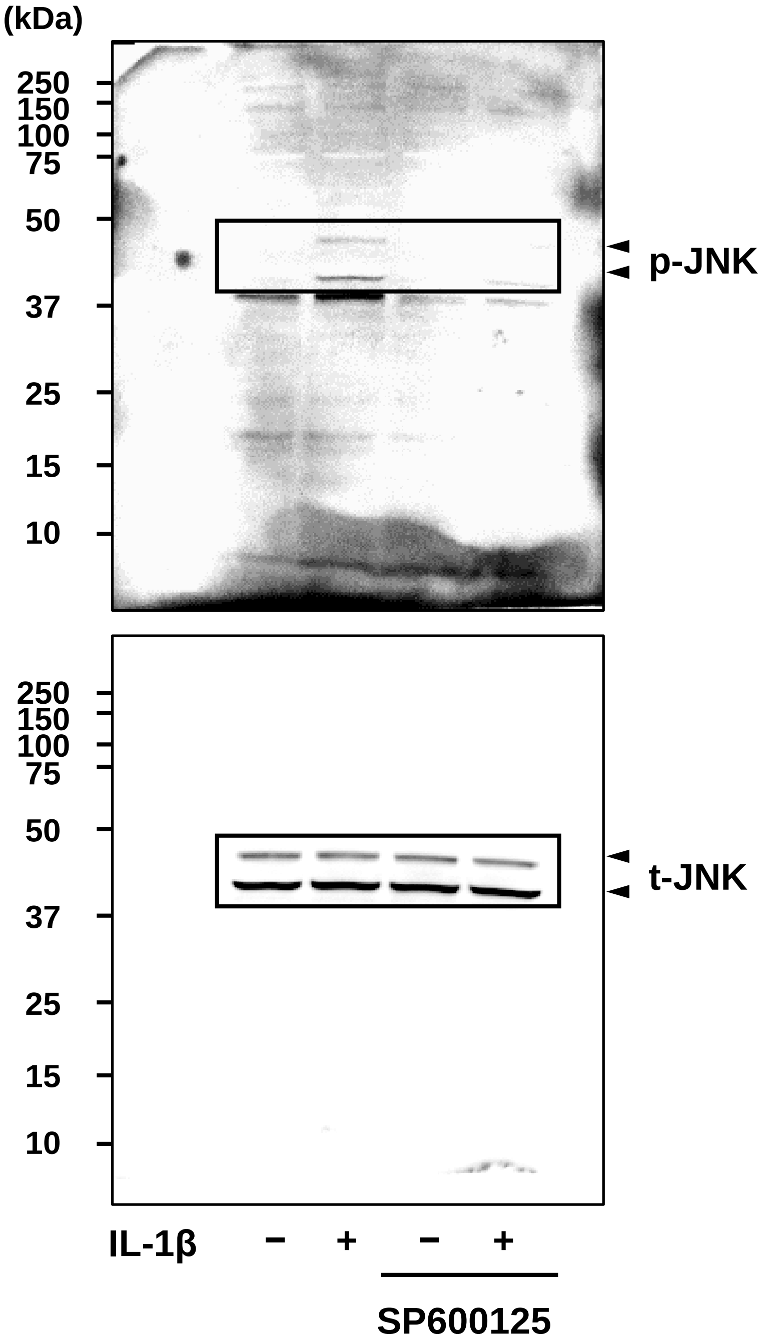

Fig. S9 Uncropped images for the blots shown in Figure 5a.

Figure 5c. original figure

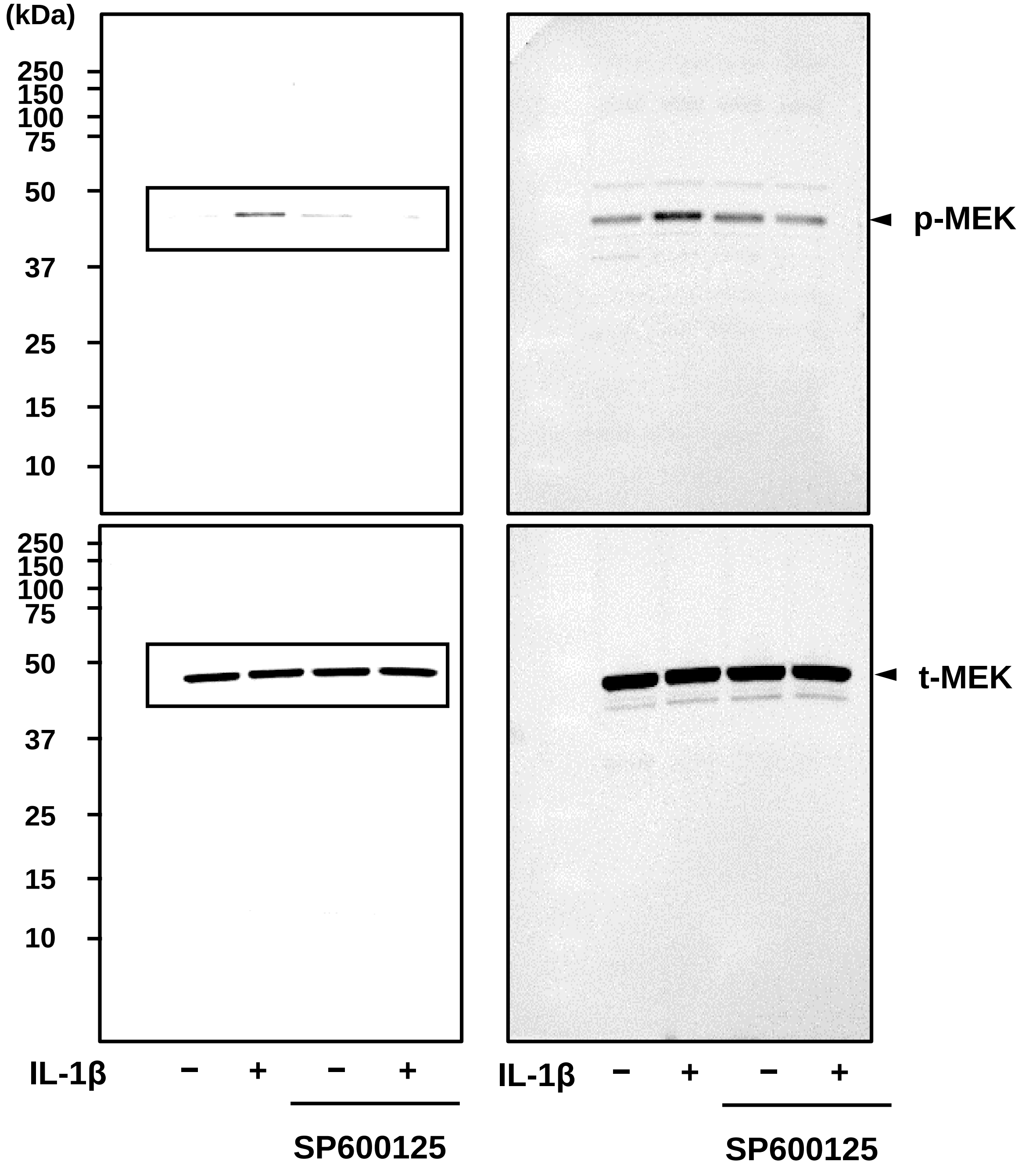

Fig. S10 Uncropped images for the blots shown in Figure 5c.

Figure 5e. original figure

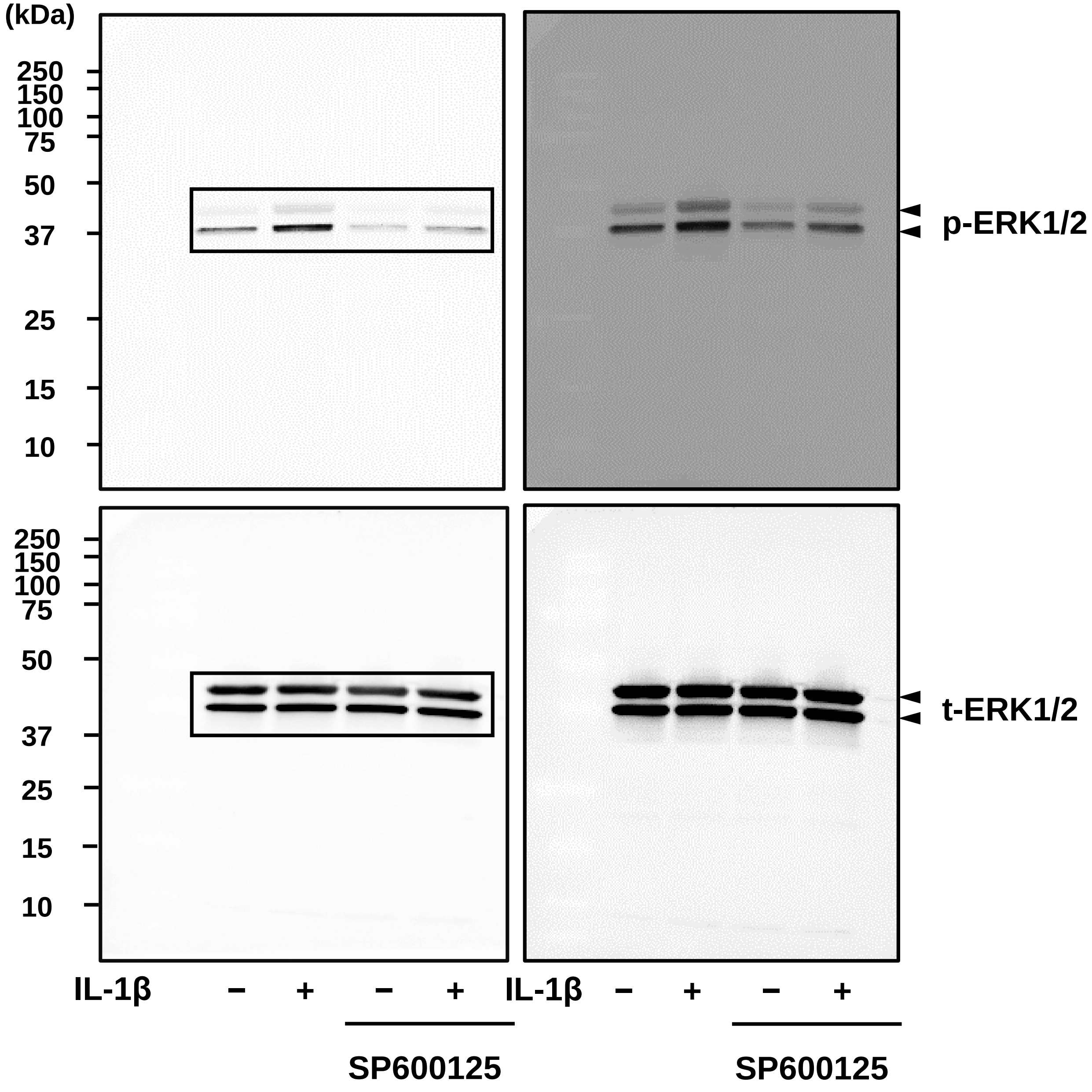

Fig. S11 Uncropped images for the blots shown in Figure 5e.

Figure 6a. original figure

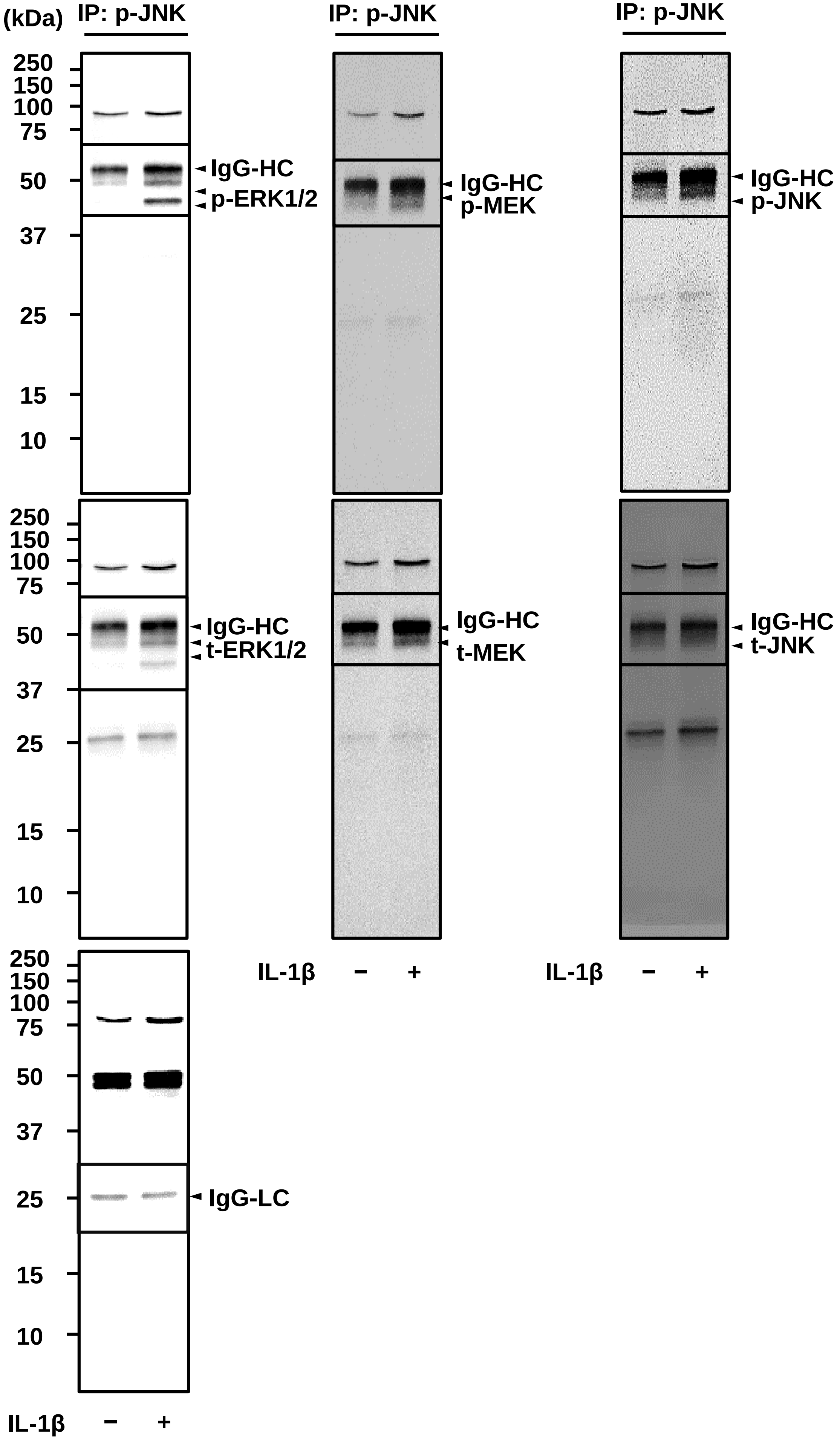

Fig. S12 Uncropped images for the blots shown in Figure 6a.

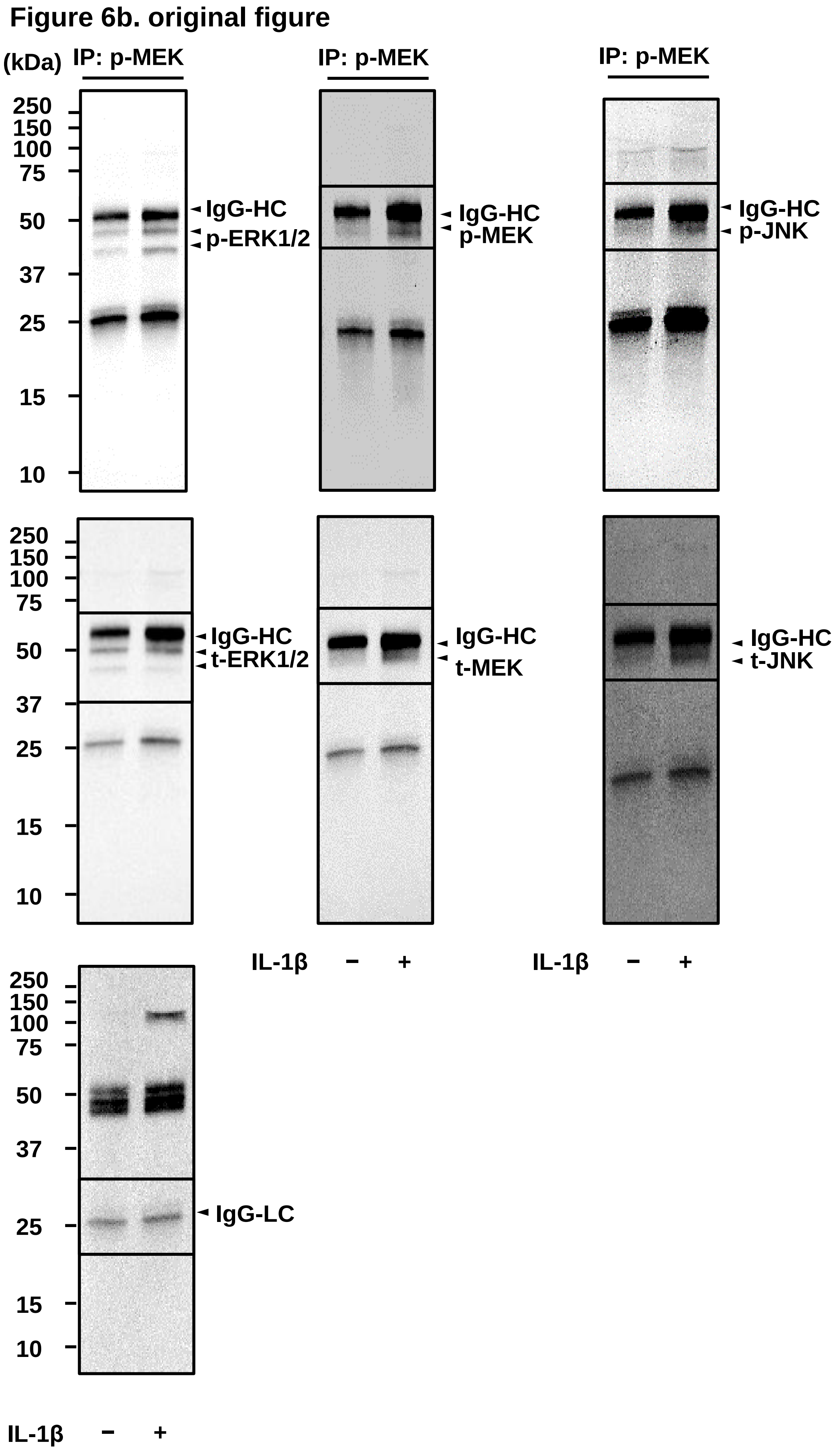

Fig. S13 Uncropped images for the blots shown in Figure 6b.

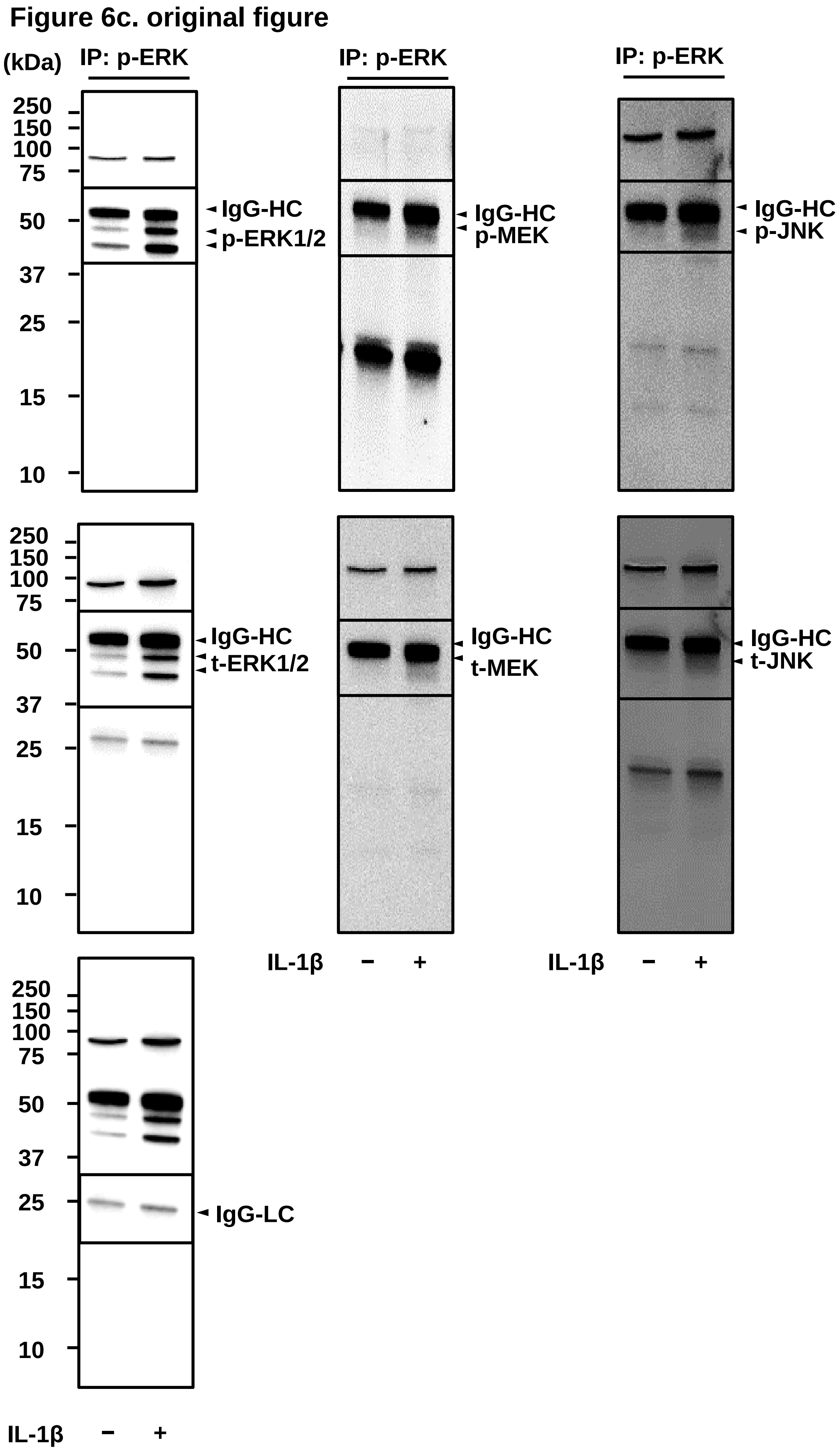

Fig. S14 Uncropped images for the blots shown in Figure 6c.

Figure 7a. original figure

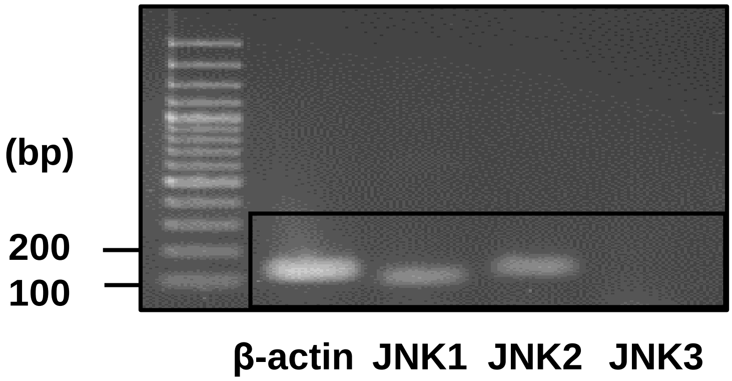

Fig. S15 Uncropped images for the blots shown in Figure 7a.

Figure 7c. first row original figure

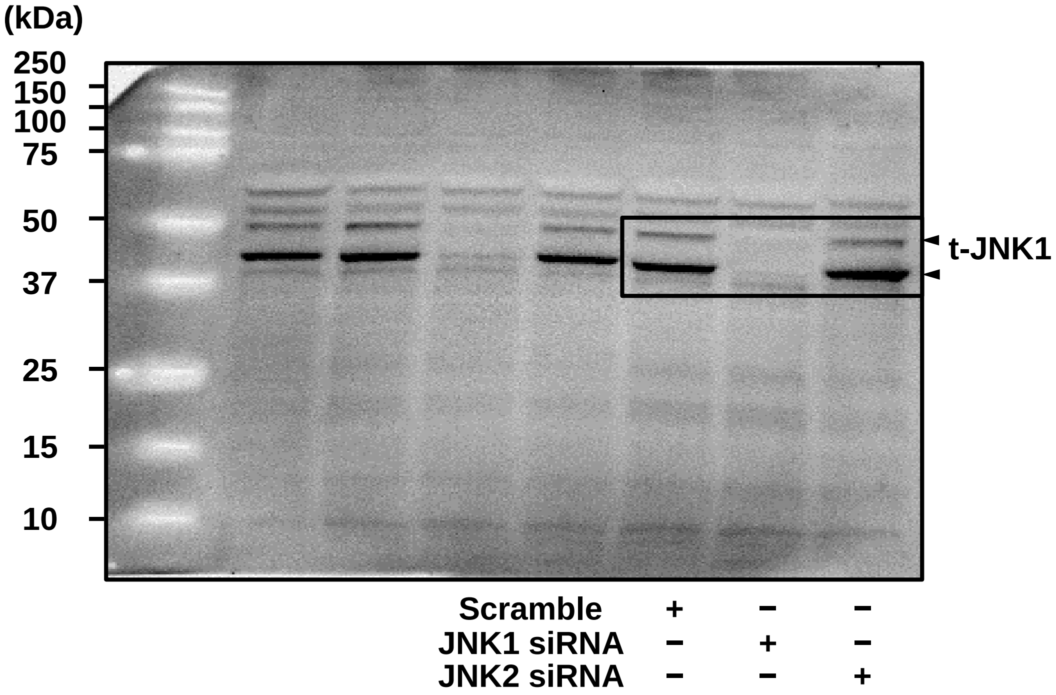

Figure 7c. second row original figure

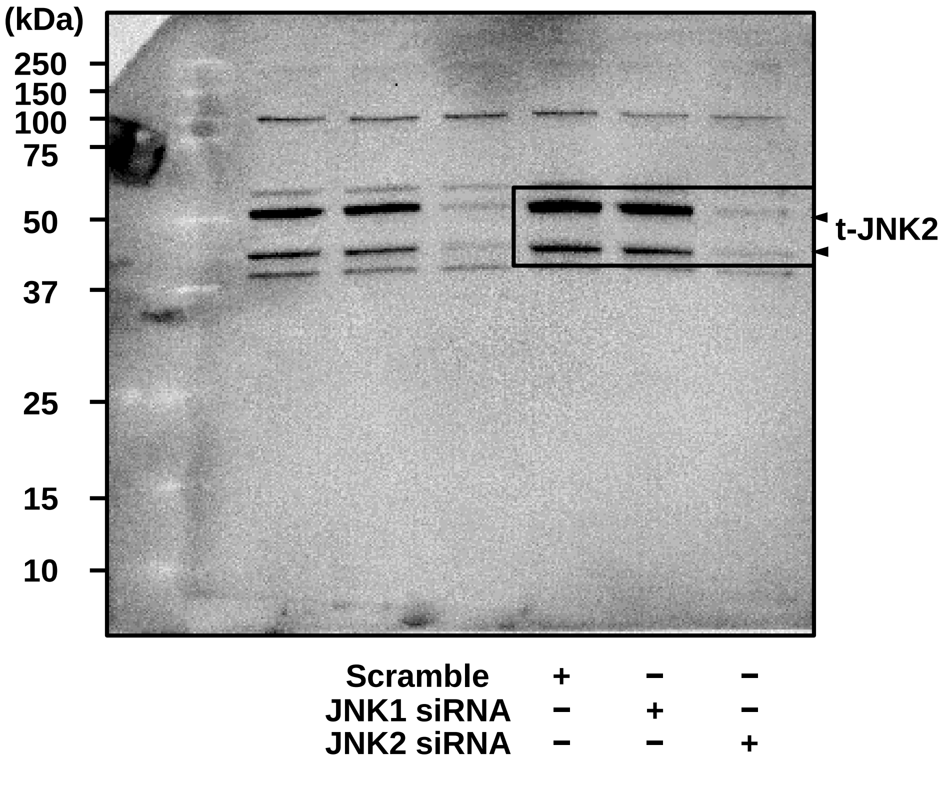

Fig. S16 Uncropped images for the blots shown in Figure 7c.

Figure 7c. third row original figure

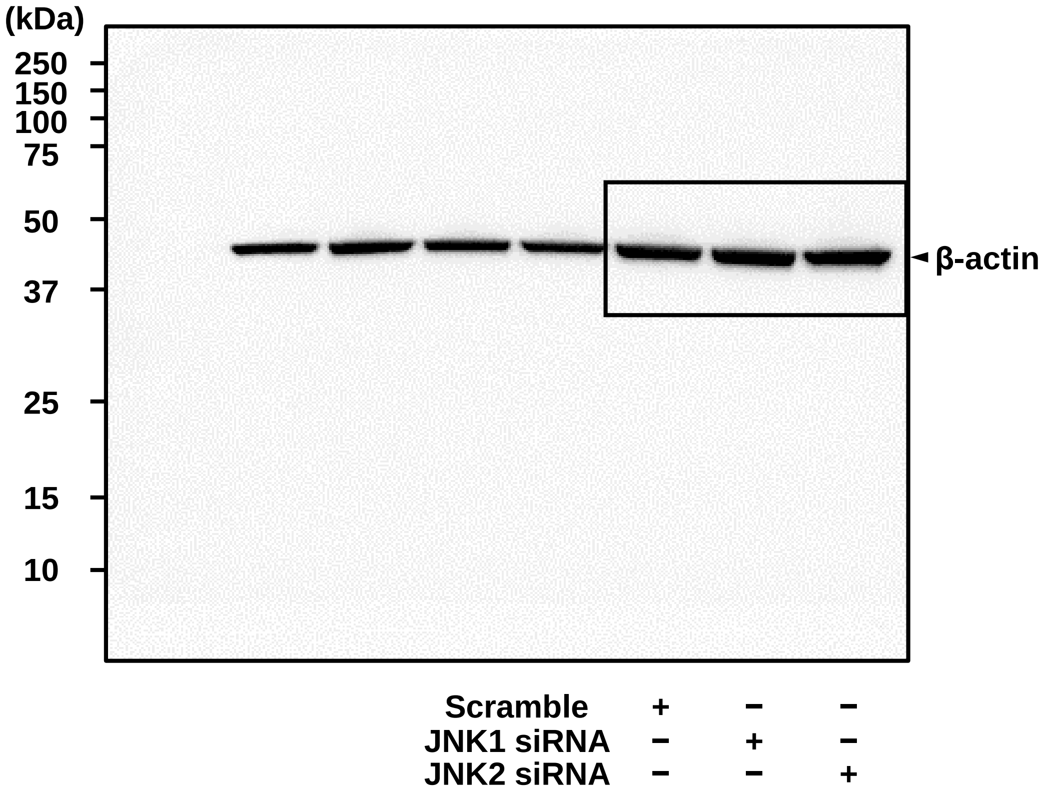

Fig. S16 (continue) Uncropped images for the blots shown in Figure 7c.

Figure 7f. first row original figure

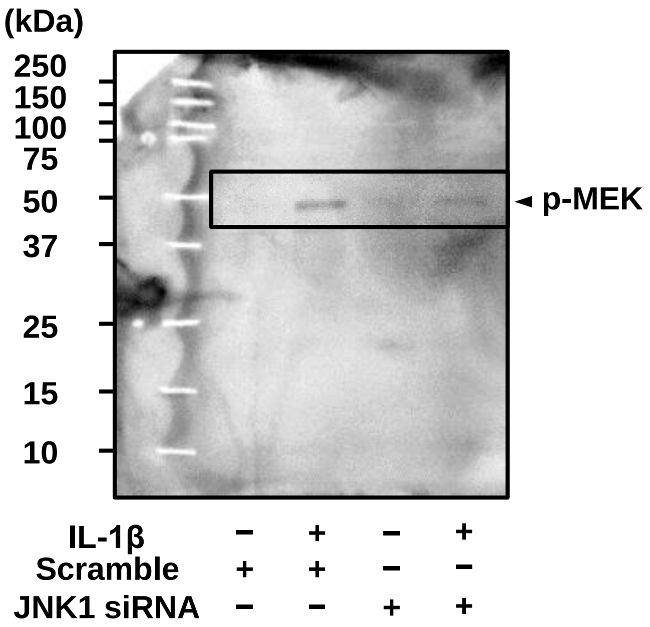

Figure 7f. second row original figure

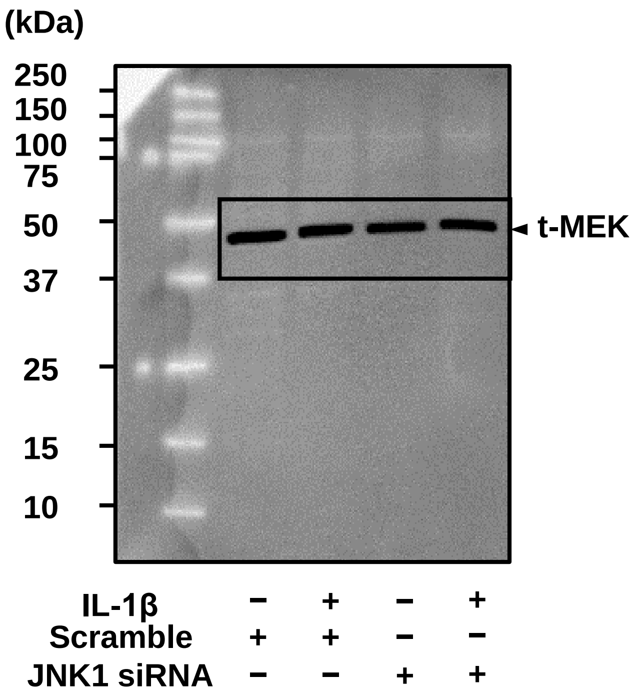

Fig. S17 Uncropped images for the blots shown in Figure 7f.

Figure 7f. third row original figure

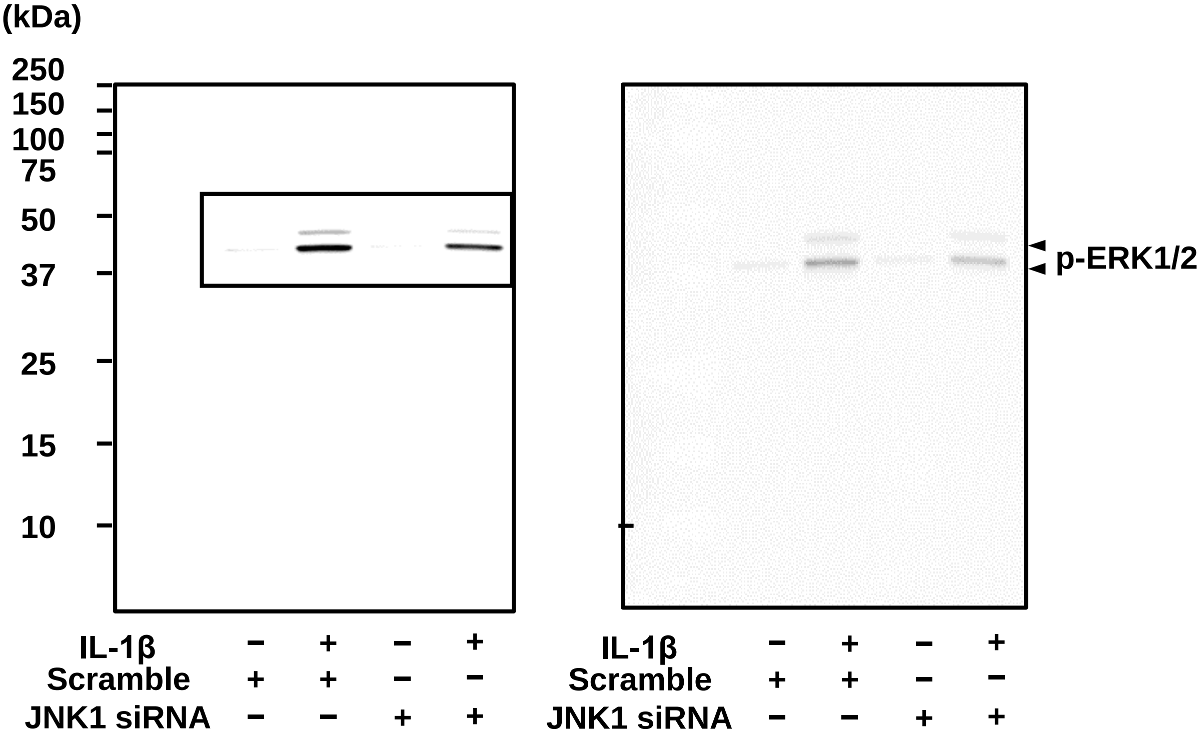

Figure 7f. fourth row original figure

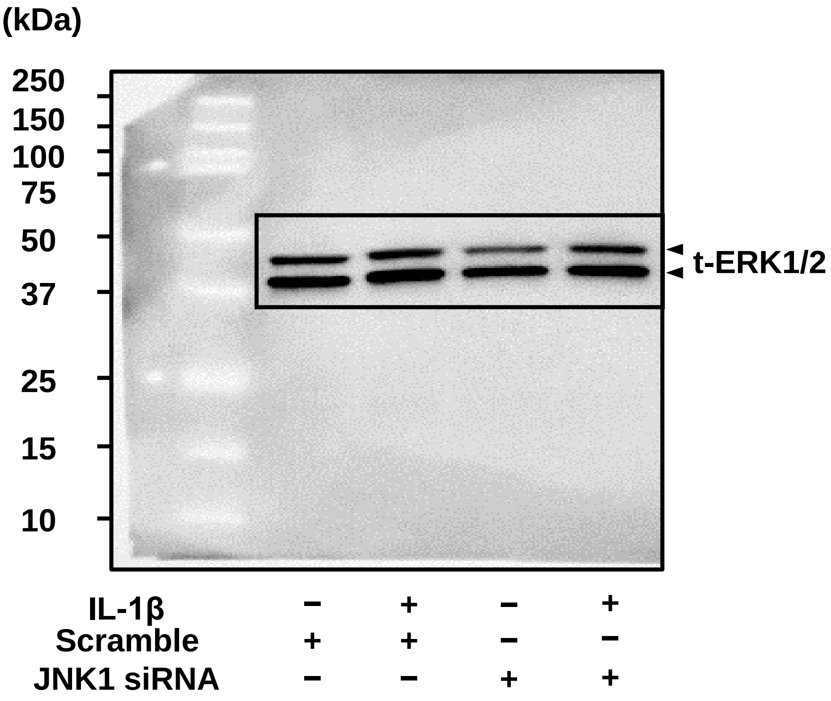

Fig. S17 (continue) Uncropped images for the blots shown in Figure 7f.

Figure 7f. fifth row original figure

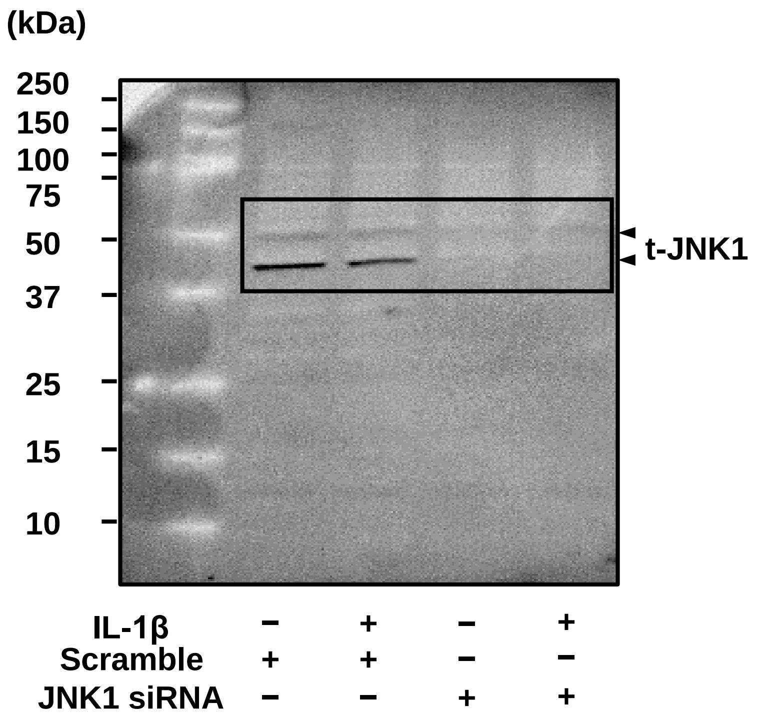

Fig. S17 (continue) Uncropped images for the blots shown in Figure 7f.

Table S1 Primers used for RT-PCR or real-time RT-PCR

Gene Name	Gene bank ID	Primer sequences
COX-1	XM_003995827	F: 5'-GGACAACCTGGAACGTCAGT-3'
		R: 5'-AAACACCTCCTGACCCACAG-3'
COX-2	EF036473.1	F: 5'-AACAGGAGCATCCAGAATGG-3'
		R: 5'-GCAGCTCTGGGTCAAACTTC-3'
JNK1	XM_011287267.1	F: 5'-AACTCTTTGACGCTGCTTGC-3'
		R: 5'-TGAAGCACTGTGCCTTTACC-3'
JNK2	XM_011281507.1	F: 5'-AGCCCAAGGGATTGTTTGTG-3'
		R: 5'-AGGACGAGTTCACGATAAGCTC-3'
JNK3	XM_011281754.1	F: 5'-TTTGTGCTGCGTATGATGCC-3'
		R: 5'-TTGGCATGCGTTTGGTTCTG-3'
β-actin	AB051104.1	F: 5'-CTCTTCCAGCCTTCCTTCCT-3'
		R: 5'- GACAGCACCGTGTTAGCGTA-3'

Table S2 Sequences for siRNA transfection

Gene Name	Gene bank ID	siRNA sequences
JNK1	XM_011287267.1	F: 5'-GAAUCAGACUCAUGCUAAA-3'
		R: 5'-TTTAGCATGAGTCTGATTC-3'
JNK2	XM_011281507.1	F: 5'-GGUAUUAUCGGGCACCCGA-3'
		R: 5'-UCGGGUGCCCGAUAAUACC-3'
